# Identification and Characterization of Differentially-Regulated Type IVb Pilin Genes Necessary for Predation in Obligate Bacterial Predators

**DOI:** 10.1038/s41598-017-00951-w

**Published:** 2017-04-21

**Authors:** Ofir Avidan, Margarita Petrenko, René Becker, Sebastian Beck, Michael Linscheid, Shmuel Pietrokovski, Edouard Jurkevitch

**Affiliations:** 1 Department of Molecular Genetics, The Weizmann Institute of Science, Rehovot, Israel; 2 Department of Plant Pathology and Microbiology, Faculty of Agriculture, Food and Environment, The Hebrew University of Jerusalem, Rehovot, Israel; 3 Department of Chemistry, Humboldt-Universität zu Berlin, Berlin, Germany

## Abstract

*Bdellovibrio bacteriovorus* is an obligate predator of bacteria that grows and divides within the periplasm of its prey. Functions involved in the early steps of predation have been identified and characterized, but mediators of prey invasion are still poorly detailed. By combining omics data available for *Bdellovibrio* and like organisms (BALO’s), we identified 43 genes expressed in *B. bacteriovorus* during the early interaction with prey. These included genes in a tight adherence (TAD) operon encoding for two type IVb fimbriae-like pilin proteins (*flp*1 and *flp*2), and their processing and export machinery. Two additional *flp* genes (*flp3* and *flp4*) were computationally identified at other locations along the chromosome, defining the largest and most diverse type IVb complement known in bacteria to date. Only *flp1*, *flp2* and *flp4* were expressed; their respective gene knock-outs resulted in a complete loss of the predatory ability without losing the ability to adhere to prey cells. Additionally, we further demonstrate differential regulation of the *flp* genes as the TAD operon of BALOs with different predatory strategies is controlled by a flagellar sigma factor FliA, while *flp4* is not. Finally, we show that FliA, a known flagellar transcriptional regulator in other bacteria, is an essential *Bdellovibrio* gene.

## Introduction

*Bdellovibrio* and like organisms (BALOs) form a unique group of gram-negative bacteria that thrive by preying on other gram-negative bacteria. They are found in diverse environments including fresh water lakes, oceans, and various terrestrial habitats^1–5^. BALOs are mainly known in the delta-proteobacteria where they form the families Bdellovibrionaceae and Bacteriovoracaceae. *Micavibrio* is the only known exception, belonging to the alpha-proteobacteria^6, 7^ Predation is either epibiotic or periplasmic. In the former, the BALO drains the cytoplasm of its prey while attaching to the prey’s outer membrane^1, 6^. In the latter, the BALO enters the prey and consumes it while residing in the prey’s periplasm. Both epibiotic and periplasmic BALOs have a life cycle that includes a highly motile small non-replicative attack phase (AP) cell, which adheres to and recognizes suitable prey; a prey envelope-derived signal then triggers a newly described transition phase during which changes in the predator and the detection a prey cytosol-associated cue^8^ leads to the cell growth phase (GP), and ultimately to cell replication. In *B. bacteriovorus*, the predator penetrates the prey’s periplasm through a transient rupturing of the prey cell wall, and remodels it into a sphere-shaped cell, the bdelloplast, by release of cell wall modifying enzymes^9^. The predator consumes the prey’s cytoplasm fueling its growth as a filamentous, multi-nucleoid cell that finally septates to form multiple progeny AP cells^9–11^.

The early steps of the predator-prey interaction involve many cell cycle-dependent, differentially expressed functions. These include expression of genes specific and indispensable for the predatory process, *i.e*. genes that are not required for intrinsic survival and growth but specific to the predatory phenotype. These genes are essential in wild type BALOs, but can be deleted in host-independent (HI) mutants^11–14^. HI mutants are generated by spontaneous mutations in the gene *bd0108*, enabling *B. bacteriovorus* to grow both on live prey cells and on rich artificial media in the absence of prey^15, 16^. Bd0108 may play a role in regulating the production and extrusion/ retraction of type IVa pilus, an appendage shown to be essential for predation in both *B. bacteriovorus* and in the epibiotic predator, *Bdellovibrio exovorus* ^17, 18^. Type IVa pili usually govern twitching motility in other bacteria^19^, but in *B. bacteriovorus* they are assumed to facilitate invasion into the prey^20^. Other predation-essential genes encoding for proteases, regulatory proteins, signal transduction related proteins and flagellar motility, and genes important for cellular structure and organization were identified by transposon mutagenesis performed in HI genetic backgrounds, combined with a screen for alterations in predatory phenotype, or by systematic homology searches^12–14, 18^.

In this study, we applied computational integrated genome and transcriptome analyses to identify genes essential for predation, based on the rationale that the AP and the earlier steps of prey invasion are unique to obligate predators. A core of 43 AP genes was identified, including genes in an operon coding for type IVb fimbriae-like protein (*flp*) genes^21^ that was further characterized. Further protein domain sequence analyses uncovered additional *flp B. bacteriovorus* genes. Genetic and phenotypic analyses showed that three of these four *flp* IVb genes are essential for predation and although they are expressed during AP, they are under different transcriptional regulation: *flp1* and *flp2* are co-regulated with flagellins by the flagella sigma factor FliA, while *flp4* is regulated independently by an unknown regulator.

## Results

### Identification of putative attack phase core genes

**Figure 1:**
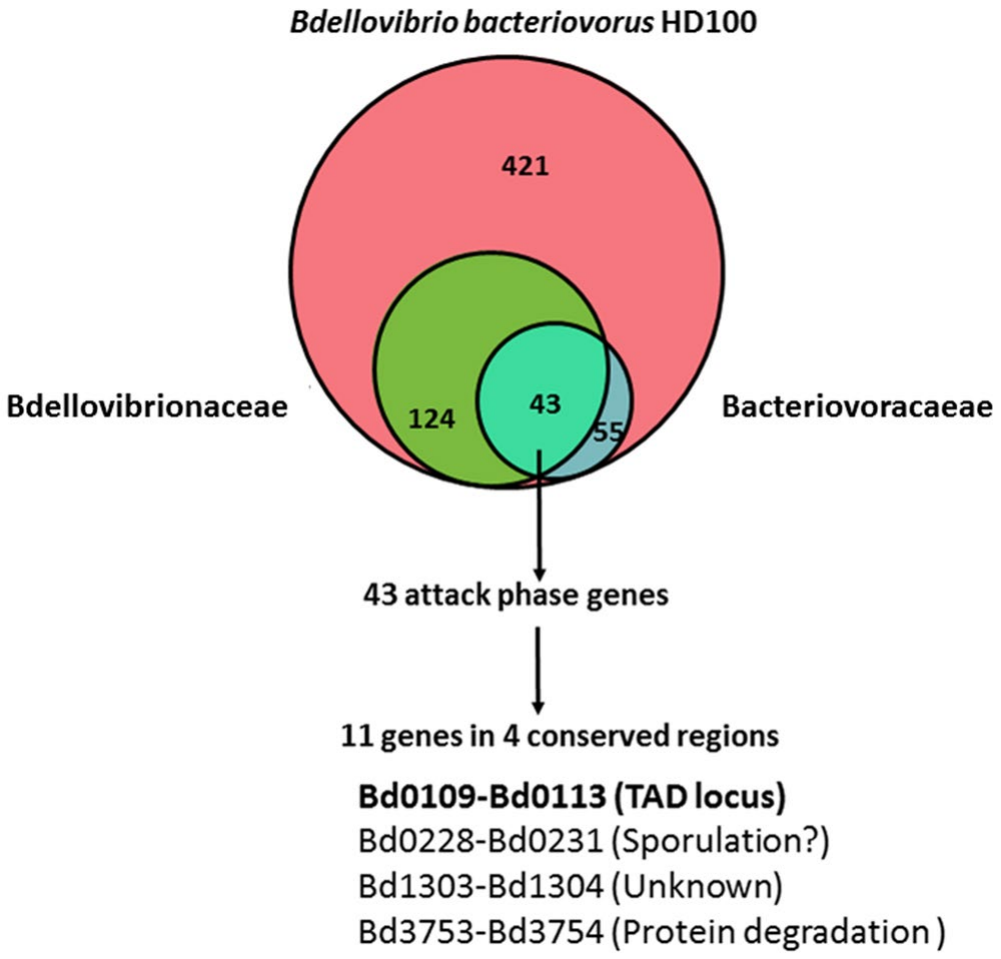
Overlap of attack phase genes between strains of *Bdellovibrio* and like organisms, identifying putative predation-essential genes. 124 Bdellovibrionaceae and 55 Bacteriovoracaceae genes exhibit high similarity (BSR ≥ 0.2) to 421 *B. bacteriovorus* HD100 AP-induced genes. The overlap of the three sets depicts 43 AP core genes, 11 of which cluster in four *B. bacteriovorus* HD100 genomic regions. Each of these regions is a putative operon as indicated by RNAseq expression data (BdelloViewer, http://www.weizmann.ac.il/molgen/Sorek/bdello_browser/viewer/index.php?meta=bdello_combined&jump=bdello,bdello_sRNAs#1 ^23^. The Venn diagram was created using Biovenn^69^.

Successful initial recognition and attachment to the prey by *Bdellovibrio* depends on factors expressed during the AP^22^. In order to identify genes that are involved in the initial step of predation, we searched for known *B. bacteriovorus* AP genes that are present in other diverse BALOs. Four hundred twenty one *B. bacteriovorus* HD100 genes that are expressed in AP^23^ were compared to three fully sequenced *Bdellovibrio* genomes (*Bdellovibrio exovorus* JSS^24^, *Bdellovibrio* sp. Arhs^25^ and *Bdellovibrio bacteriovorus* W (https://www.ncbi.nlm.nih.gov/assembly/GCA_000525675.1), three fully sequenced *Bacteriovorax* strains (*Bacteriovorax* SEQ25_V, *Bacteriovorax* BAL6_X, *Bacteriovorax* BSW_11IV^4^ and the fully sequenced *Halobacteriovorax marinus* SJ^26^. *B. bacteriovorus* HD100 and *B. bacteriovorus* W are periplasmic fresh water BALOs, *Bdellovibrio* sp. ArHS is a thermophilic BALO, and *B. exovorus* JSS is a fresh water epibiotic predator within the *Bdellovibrionaceae*. *H. marinus* SJ and the three *Bacteriovorax* strains are marine periplasmic BALOs belonging to the Bacteriovoracaceae^1, 4, 25, 26^. Forty-three genes that are certain orthologs between the examined species that had high (0.2) BLAST score ratios (BSR) were identified in this computational screen. To further refine our screen, looking for additional evidence for evolutionary conserved genes^27^, we searched the 43 “AP core” gene set for members that are syntenous in all examined BALOs. Twelve of the 43 genes were found to be syntenous and present in four putative operons (Fig. 1 and Table S1).

Most of the genes in the four putative operons encode for poorly characterized or unknown proteins. Genes *bd0228* to *bd0231* form a putative operon that encodes a serine protein kinase, an uncharacterized protein and a sporulation protein R, SpoVR. Similar operons were found in many non-sporulating and non-predatory bacteria from the beta-, gamma- and delta-proteobacteria. SpoVR is a non-essential gene that is involved in spore cortex formation in *Bacillus subtillis* and *Myxococcus* *xanthus* ^28, 29^. Genes *bd1103* and *bd1104* are in a putative operon and encode two conserved proteins of unknown function, also found in many bacteria. The putative operon *bd3753*–*bd3754* encodes for ClpX and ClpP proteins respectively, forming the ClpXP complex that degrades misfolded proteins and thus assists in maintaining cell proteostasis^30^. Interestingly, the *E. coli* ClpXP complex is activated under carbon starvation, the physiological state of the attack phase^31, 32^.

The syntenous region *bd0109*–*bd0113* is part of a putative operon including genes *bd0109*–*bd0119*. It was previously annotated as a tight adherence locus (TAD)^33^. This operon encodes for proteins that process and export fimbriae like proteins (Flp) to the outer membrane. Flp proteins were found to be crucial for bacteria-host interaction in human, animal, and plant pathogens^34, 35^.

### *B. bacteriovorus* HD100 genome encodes four different flp pilin genes

**Figure 2:**
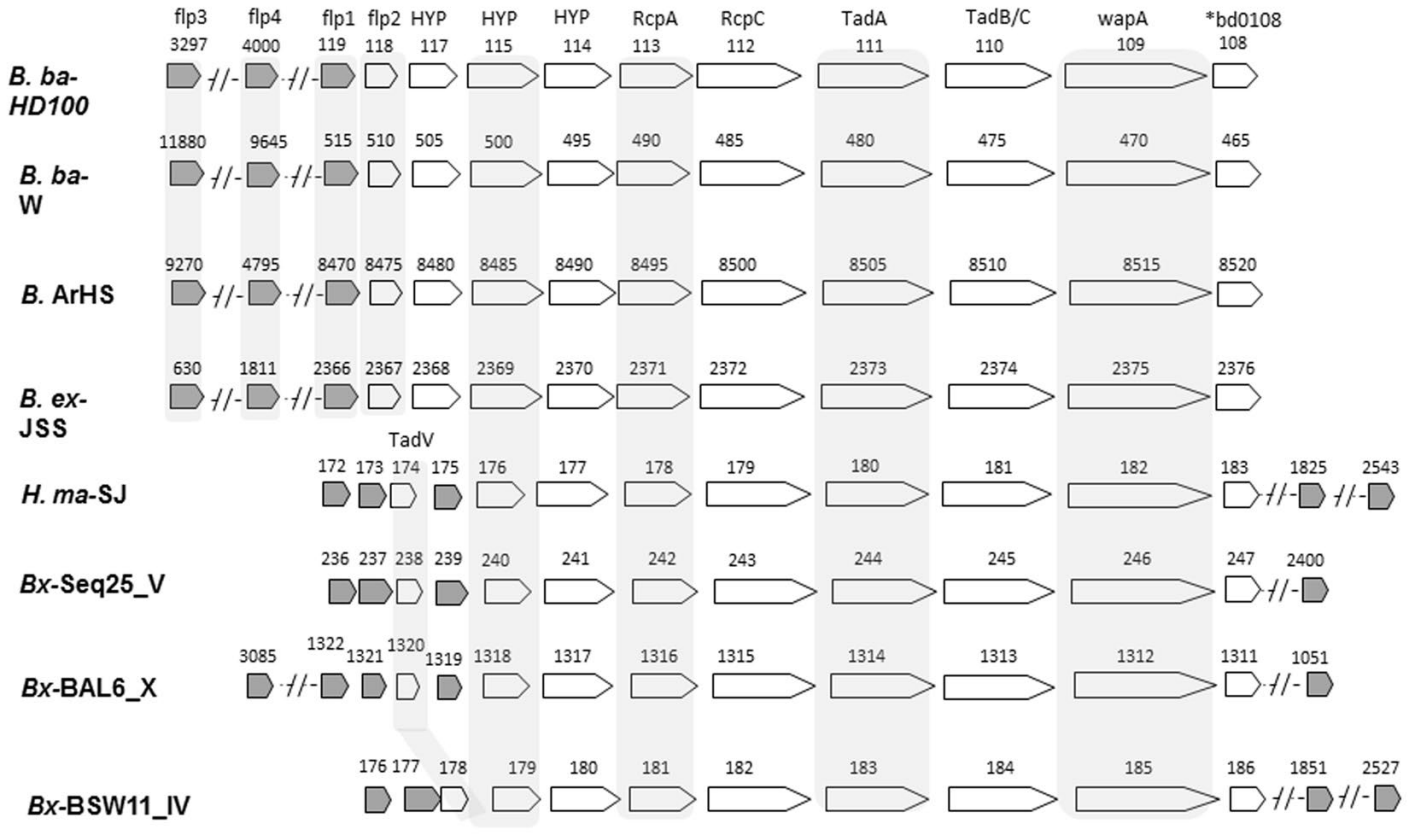
Genomic arrangement of TAD loci in different BALOs. TAD loci from all examined BALOs exhibit synteny. The prepilin peptidase, TadV is missing from all *Bdellovibrio* genomes but is present in *Halobacteriovorax* and *Bacteriovorax* genomes. All BALOs also encode non-TAD associated *flp* genes. *flp*2 is a TAD-associated trimmed *flp*, that is only present in *Bdellovibrio* TAD loci and is absent from all other BALOs. BALO species designations- B.ba-HD100: *Bdellovibrio bacteriovorus* HD100; B.ex-JSS: *Bdellovibrio exovorus* JSS; B.ba-W: *Bdellovibrio bacteriovorus* W; B-ArHS: *Bdellovibrio* sp. ArHS; H.ma-SJ: *Halobacteriovorax marinus* SJ; Bx-SEQ25_V: *Bacteriovorax* sp. Seq25_v; Bx- BAL6_X: *Bacteriovorax* sp. BAL6_X; Bx- BSW11_IV: *Bacteriovorax* sp. BSW11_IV.

The TAD locus operon encodes components essential for the maturation and export of Flp proteins^34^. The region includes genes coding for the ATPase that drives Flp export, TadA (*bd0111*); an inner membrane export component, TadB (*bd0110*); the outer membrane export proteins RcpA (*bd0112*) and RcpC (*bd0113*)^33, 36^. However, TadV, a prepilin peptidase crucial for pilin maturation, is missing from the operon and was not found in the entire *B. bacteriovorus* genome. Further analysis indicates that TadV is missing from all known *Bdellovibrio* genomes, but is present in *Halobacteriovorax* and all of the *Bacteriovorax* genomes (Fig. 2).

**Figure 3:**
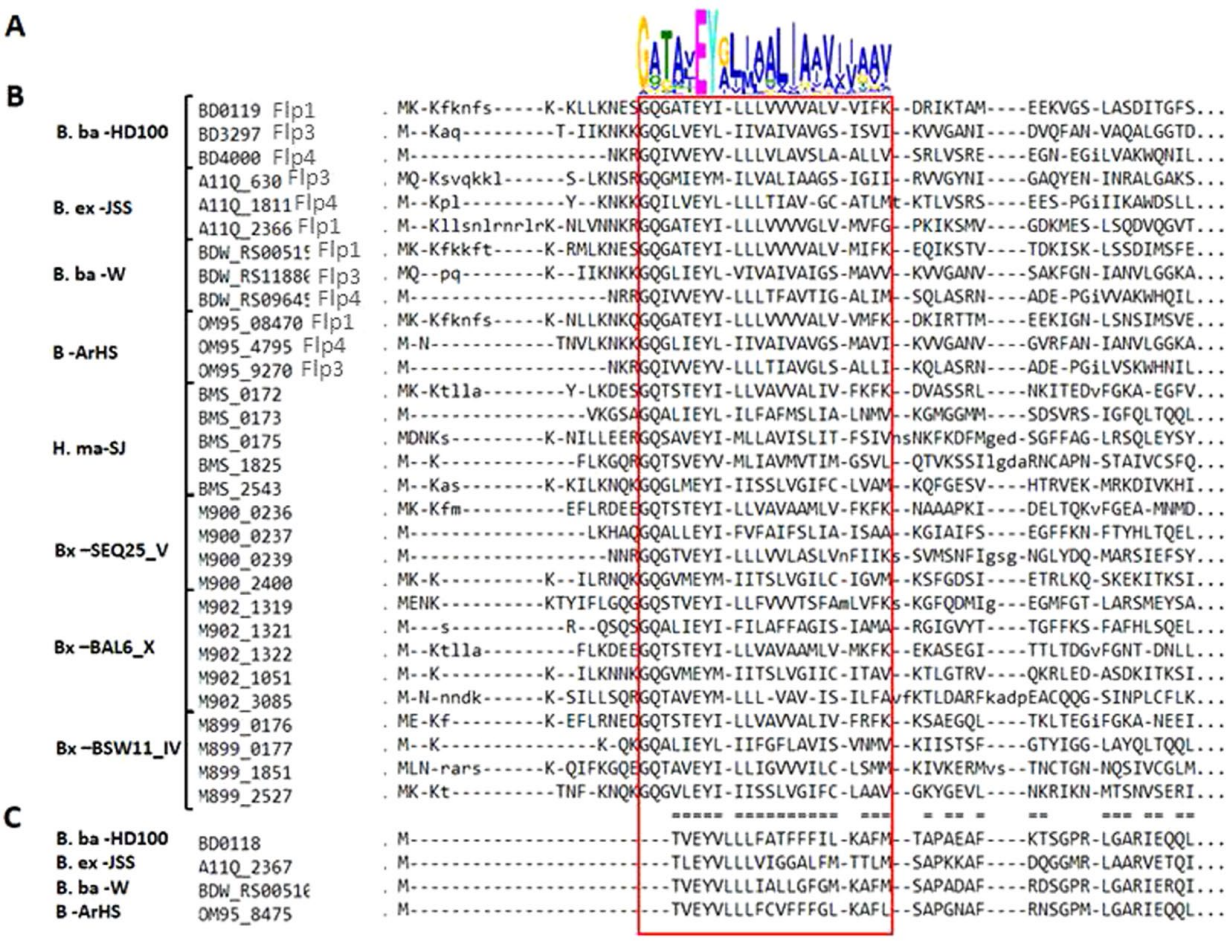
Multiple alignment of predicted *flp* sequences. Sequence logo representation representation^70^ of a Flp pilin sequence position weight matrix (PWM), depicting the canonic Flp pilin motif (GXXXXEY and a stretch of hydrophobic amino acids). (**A**) Multiple alignment of Flp pilin sequences identified by the PWM in various BALO’s with the *flp* pilin motif indicated by a red outline. (**B**) Flp2 sequences are truncated forms of Flp pilins, both the N terminus proximal glycine (G) and the amino acid that follows it are missing, creating motif: MXXEY and a stretch of hydrophobic amino acids. The protein logo was created with MEME^60^. Multiple alignments were calculated using GLAM2^60, 71^. The Flp and the Flp2 multiple alignments were aligned to each other using the Compass program version 2.45^72^, with the similar regions indicated by the ‘=’ marks in between the multiple alignments. Lower case letters are regions not found to be significantly aligned by GLAM2. BALO designations are as in Fig. 2.

Based on their genomic position within the TAD locus, their small size, and the hydrophobic character of the encoded proteins, two *B. bacteriovorus flp* pilin protein-encoding genes were previously identified (*bd0119*, *flp*1, and *bd0118, flp*2)^33^. We searched BALOs genomes for additional *flp* pilin genes, encoded outside the TAD locus. Flp pilins are short and not well conserved^37^. We thus searched not with specific *flp* pilin sequences (*i.e*., by BLAST searches) but with conserved amino acid motifs. We first constructed a position weight matrix (PWM) of conserved motifs from different Flp pilin protein sequences (Experimental procedures, Fig. 3). The PWM corresponded to a known conserved Flp proteins sequence domain, a GXXXX﻿EY followed by a stretch of hydrophobic amino acids^37^. Searching the *B. bacteriovorus* genome with the PWM identified three putative *flp* pilin encoding genes: *bd0119* (*flp1*), *bd3297* (*flp3*) and an un-annotated open reading frame within the *bd2719*–*bd2720* intragenic region that we designated as *bd4000*.

Searching other BALO genomes with the PWM revealed that all include similarly located *flp* pilin open reading frames, i.e. *flp* sequences in the TAD locus and others that are not TAD associated (Fig. 3, Fig. S1). While only one TAD-associated *flp* gene was found with the PWM in *Bdellovibrio* genomes, *Bacteriovorax* and *Halobacteriovorax* genomes include three TAD associated *flp* pilin genes. Interestingly, *bd0118*, annotated as *flp2* in *B. bacteriovorus* ^33^ was not identified as a Flp pilin-encoding gene by our PWM. Sequence analysis demonstrated that Flp2 includes a trimmed Flp domain. The Flp*2* sequence starts with MXXEY that is followed by a stretch of hydrophobic amino acids (Fig. 3). This domain differs from the consensus Flp domain as it misses four amino acids: the glycine residue that is recognized by the prepilin peptidase and the three residues C-terminal to it that typically are at the N-terminal end of the processed, mature pilin^38^. Consequently, while the Flp motif is usually 5–20 amino acids from the protein initiator methionine, in Bd0118 it directly follows it (Fig. 3). Genes encoding proteins with such a trimmed Flp motif were also observed within the TAD loci of other *Bdellovibrio* strains, but not in *Bacteriovorax* (Figs 2 and 3).

In order to exclude the possibility of mis-annotation of the coding region start position, we added its upstream 21 amino acids to the Bd0118 sequence and compared it to the PWM. No complete *flp* motif was found in Bd0118 or its orthologs in other BALO genomes. *flp3* and *flp4* were annotated as Flp proteins in additional *Bdellovibrio* strains based on the synteny of their surrounding genes with those of the *B. bacteriovorus* HD 100 *flp3*, and *flp4* genes (Table S2).

A similar screen in four Bacteriovoracaceae genomes identified three TAD-associated and three non-TAD- associated *flp* pilin genes in *H. marinus* SJ, *Bacteriovorax* Seq25_V and *Bacteriovorax* BAL6_X. *Bacteriovorax* BSW11_IV was the only exception as it included two *flp* genes from each kind.

### *flp1*, *flp2*, and *flp4* are expressed during attack phase while *flp3* is silent

**Figure 4:**
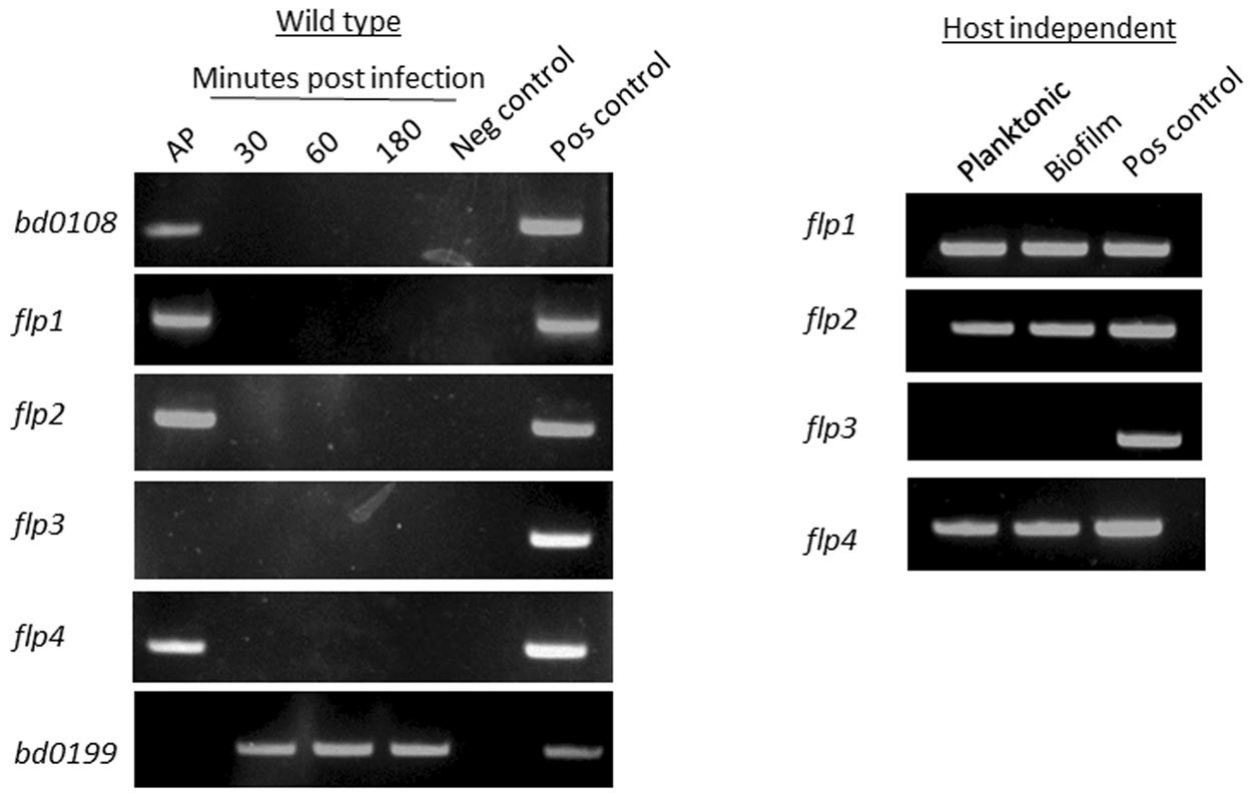
Semi-quantitative RT-PCR (sqRT-PCR) of *B. bacteriovorus flp* pilin genes. Expression of *flp* pilin encoding genes during the *B. bacteriovorus* HD100 life cycle shows that *flp1, flp2* and *flp4* are expressed during AP, while *flp3* is silenced during the whole cycle. The same expression pattern was observed in a host-independent biofilm and in a planktonic culture, with *flp1* and *flp2* are expressed and *flp*3 is silent. *bd0108* is used as a AP marker and *bd0199* as a GP marker. A) Negative control - *E. coli* DNA. B) positive control - *B. bacteriovorus* DNA. Gels were acquired with DNR Gel Capture with no further processing.

The expression pattern of the four *flp* genes were analyzed in AP, and at early (30 min), mid (60 min), and late (180 min) GP. *flp1*, *flp2*, and *flp4* were only expressed in the AP, while *flp3* was silent at all examined time points (Fig. 4). *flp* gene expression was further examined in the planktonic and biofilm phases of HI mutant cultures. *flp1*, *flp2*, and *flp4* were expressed in both planktonic and biofilm cultures, while *flp3* was silent under both conditions (Fig. 4). Gene *bd0199*, which is only expressed during GP, served as a control. A protein analysis by mass spectrometry (MS) of wild type *B. bacteriovorus* cultures in which Bd0110, Bd0111, Bd0112, Bd0113 and the Bd0119 (Flp1) were detected, confirmed that TAD operon-encoded proteins were indeed produced in AP (Table S3).

### *flp1*, *flp2*, and *flp4* pilins knockouts abolish predation

**Figure 5:**
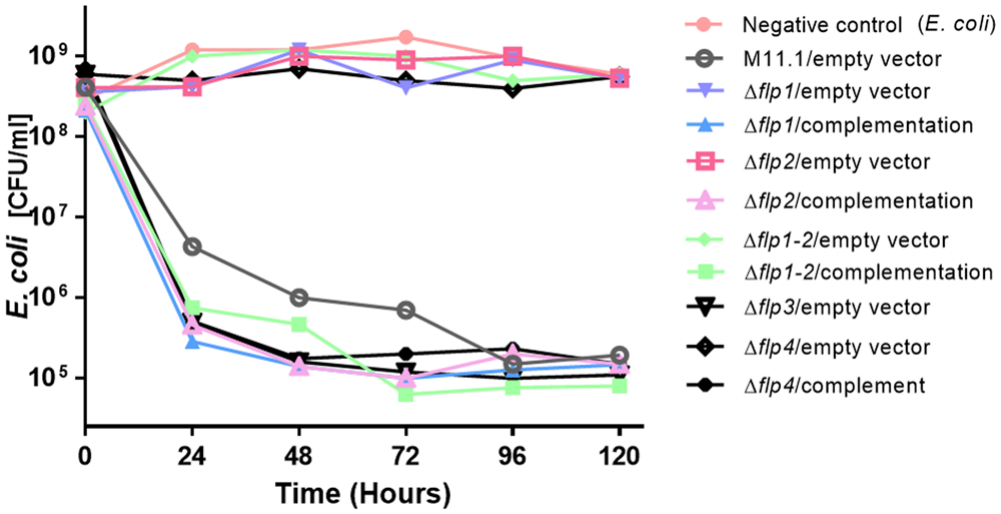
Effects of *flp* pilin gene deletion on predation. Both TAD-associated *flp1, flp2*, and non-associated *flp4* gene knockouts (Δ*flp1*, Δ*flp2*, and Δ*flp4*, respectively) are unable to prey, while Δ*flp3* had no effect on predation. All strains regained the predatory phenotype of the parental strain (M11.1) upon complementation (comp.Δ*flp1*, comp.Δ*flp2* and comp. Δ*flp4*). In order to prevent polar effects, all knockouts were constructed as in-frame deletions, with only small non-functional peptides encoded in the remaining sequence (9–12 amino acids long). Complementation for each mutation was carried out using pPROBE-NT. Control strains carried an empty vector.

In order to examine the role of Flp pilins-encoding genes in predation, all four *flp* genes were subjected to in-frame deletion in the background of *B. bacteriovorus* HD100. While *flp3* (which was not expressed under any of the examined conditions) could be deleted in this wild-type background, meaning the mutated strain could grow predatorily, *flp1*, *flp2* and *flp4* could not be deleted, as a screen of about 400 plaques of each for putative double recombinants did not yield any. For comparison, the *flp3* deletion mutants were identified at a frequency of one per 20 plaques screened with a confirmation rate of 60%. This strongly suggests that both the typical *flp1* and *flp4* type IVb pilin genes, and the *flp*2 truncated Type IVb pilin gene, are essential for predation in a wild type background. To examine this hypothesis, these *flp* genes were deleted separately and also together in the background of HI M11.1, a *B. bacteriovorus* HD100 spontaneous host-independent type II mutant^16^. This mutant can grow axenically, in the absence of prey but retains predatory capabilities, albeit with reduced efficiency. This yielded the double mutant HIΔ*flp1-2*, the triple mutant HIΔ *flp1-2*-4 and the three single mutant strains HIΔ*flp1*, HIΔ*flp2*, *and* HIΔ*flp4*. These five mutant strains grew axenically, *i.e*. in the absence of prey on a rich PYE medium as expected from HI strains. However, none was able to prey on living *E. coli* cells, demonstrating their indispensability in the early stages of the predatory interaction (Fig. 5, Fig. S2). Finally, to confirm that the predation inability of these deletion strains was caused by the introduced deletions, the strains were complemented with the deleted genes under their native promoter by conjugating plasmids pPROBE-NT-*flp1-2*, pPROBE-NT-*flp1*, pPROBE-NT-*flp2* and pPROBE-NT-*flp4* each to its corresponding deletion strain. Complementation resulted in complete restoration of the predatory HI phenotype (Fig. 5, Fig. S2), causing a four-order of magnitude decrease of the prey population. Deletion of *flp3* in the HI M11.1 background did not yield any noticeable phenotypic change, confirming that, as in the wild type background and under laboratory conditions, *flp3* is not required for predation. To address the possibility of functional complementation between *flp* genes, plasmid pPROBE::*pflp1-flp3*, bearing *flp3* under the *flp1* promoter was introduced in the HIΔ*flp1*and in the HIΔ*flp4* backgrounds. No complementation was observed, as predation was not restored.

### *flp1*, *flp2*, and *flp4* do not mediate binding to biotic or abiotic surfaces

**Figure 6:**
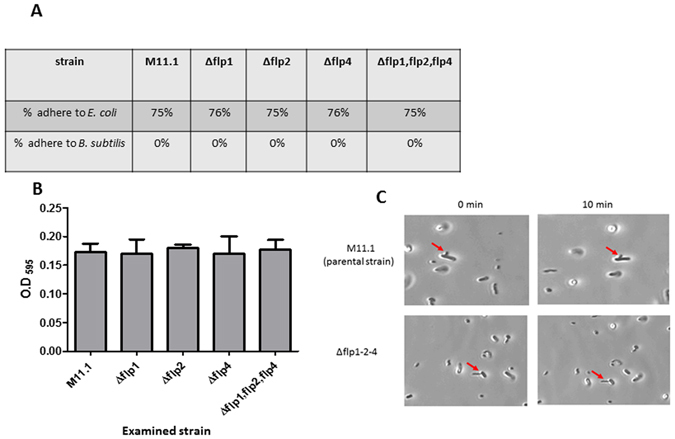
Adherence of *flp* knockouts to live prey and to abiotic surfaces. (**A**) HI M11.1 single and triple knockouts of *flp1, flp2* and *flp4* were examined for their ability to adhere to *E. coli* cells. No difference was observed between all the knockout strains and the HI M11.1 parental strain. *B. subtilis* served as a negative control for adhesion. (**B**) Examination for biofilm formation (adherence to abiotic surfaces), as in section A, no difference was observed between the strains. (**C**) Phase microscopy illustrating the adhesion assay. As in the parental HI M11.1 strain, the non-predatory mutant Δ*flp*1-2-4 only attached to *E. coli* through the predatory pole. Similar results were obtained with the individual *flp1*, *flp2* and *flp4* mutants (>100 events examined), in suspensions containing 10^6^ or 10^7^ cells.ml^−1^. In all mutants, the cells did not detach from *E. coli* during the examination time (10 min). Pictures were taken with a Digital Sight D5 camera (Nikon) and captured with a NIS-element software with no further processing.

We examined if the non-predatory phenotype of the *flp* deletions is due to a reduced capacity for surface adhesion, as is the case in other bacteria where *flp* genes mediate surface attachment^39–45^. For that purpose, biofilm formation by the parental HI strain, M11.1 and the type IVb pilins triple knockout strain ∆*flp1-2*-4, was examined. No significant reduction in biofilm formation was observed (Fig. 6).

The single *flp1*, *flp2*, and *flp4* mutants as well as the triple *flp1-2*-4 knockout were then examined for direct binding to *E. coli* prey cells. No significant difference to the parental strain M11.1 was evident (Fig. 6). Direct observation under phase contrast microscopy showed that the Δ*flp*1-2-4 mutant as well as each single *flp* mutant can bind to prey cells for significant lengths of time (>10 min).

### The *B. bacteriovorus* HD100 and *B. exovorus* JSS TAD operons are regulated by the flagellar sigma factor FliA

A transcriptomic analysis of *B. bacteriovorus* AP and GP^23^ suggested that the flagellar sigma factor, *fliA* (*bd3318*) acts as the main regulator of AP-expressed genes, including those encoded in the TAD operon. To demonstrate FliA control over TAD, we attempted to delete the *B. bacteriovorus* sigma factor FliA, encoded by gene *bd3318*. No mutants were obtained after screening 300 plaques of wild type HD100 and 300 colonies of a HI derivative. We then focused our efforts on the HI strain for technical convenience. As FliA is a central regulator of a cell cycle phase, we conjectured that a *fliA* knockout would be lethal or grow slowly. Accordingly, the knockout procedure was modified (see experimental procedures). No mutants were uncovered under any of the incubation conditions employed: colonies that appeared after 2 to 3 weeks of incubation were all found to be wild type. When low, 2% and 3% sucrose solutions were used for counter-selection, 30 to 50% of the colonies were merodiploid. No full recombinant was obtained. Therefore, the inability to knockout *fliA* led us to the conclusion that FliA is essential in *B. bacteriovorus*, unlike what was found in other bacteria^46^.

**Figure 7:**
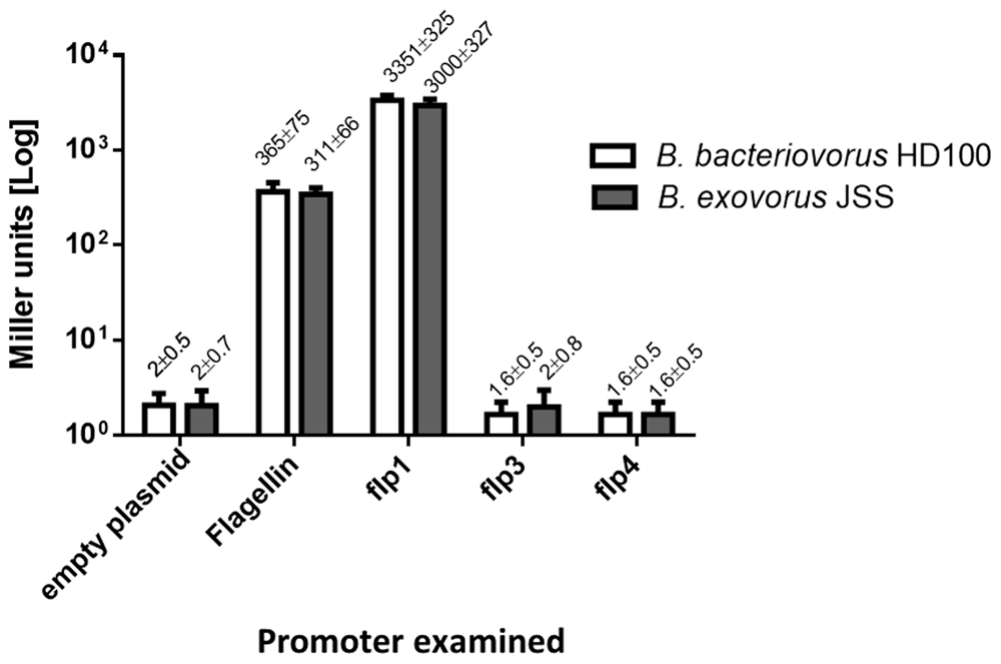
Examination of *B. bacteriovorus* and *B. exovorus* FliA-dependent promoter activity in an *E. coli* heterologous system. *B. bacteriovorus* HD100 and *B. exovorus* JSS FliA (*bd3318* and *a11q_617*, respectively) activity was examined in an *E. coli* heterologous system. Flagellin promoters (*Bd0606, A11q_1936*) and an empty vector were used as positive and negative controls, respectively. The promoters of *flp1* genes (*bd0119* and *A11q_2366*) responded strongly to FliA induction in comparison to the controls. In contrast, promoters of the  *flp3* (*bd3297* and *a11q_630*) and *flp4* (*bd4000* and *a11q_1811*) genes did not respond to FliA induction.

The *flp*1 promoter (i.e., promoter of the TAD operon) (Fig. 7, Fig. S3) was examined in a heterologous *E. coli* system: *fliA* coding regions *(bd3318* or a11q_0617 for *B. bacteriovorus* and *B. exovorus*, respectively) were cloned into pBAD/gIIIA under an arabinose inducible promoter. Since the examined promoters were cloned upstream of a promoter-less β-galactosidase (see experimental procedures), recognition of these promoters by a sigma factor, such as FliA, should result in a measurable β-galactosidase biochemical activity. In other gram-negative bacteria, flagellin-encoding genes are regulated by FliA^46^, as was also predicted for *B. bacteriovorus* ^11, 23^. Therefore, *B. bacteriovorus* HD100 and *B. exovorus* JSS flagellin *(bd0606* and *a11q_1936* respectively*)* promoters were chosen as positive controls. β-galactosidase under the TAD operon (that includes *flp1* and *flp2*) or under the regulation of ﻿﻿﻿﻿flagellin promoters was strongly induced by FliA, as compared to an empty pBBR1-MCS2-LacZ vector. This validated the hypothesis that FliA regulates expression of *flp*1 and ﻿of﻿ all  the other AP genes present with it in the TAD operon (Fig. 7). In contrast, the *flp*3 and *flp*4 promoters from both *B. bacteriovorus* HD100 and *B. exovorus* JSS remained silent under these conditions (Fig. 7).

## Discussion

Genes essential for predation by *B. bacteriovorus* have been previously identified by direct random transposon mutagenesis, screening for mutants defective in predation^12, 13^, or by targeting *B. bacteriovorus* genes whose orthologs in other bacteria encode functions that could potentially affect predation^14^. In this work, we aimed to identify genes that are essential for predation by examining early predation stages of the *B. bacteriovorus* life cycle. Genomic data have so far been used to differentiate between predatory and non-predatory bacteria, between predators exhibiting an epibiotic versus a periplasmic strategy, and to delineate the core genes of periplasmic predators^4, 24, 26, 47^. By integrating BALOs genomic and transcriptomic data we identified a set of 43 genes designated as “attack phase core genes”.

We focused on the TAD operon (*bd0109-0119*), that encodes *flp* (type IVb) pilin-encoding genes and their processing and export machinery. Our analyses showed that type IVb pilin genes, while found in various gram negative bacteria, are more numerous in BALOs than in other known bacteria and that they are very diverse in sequence, in their association with the TAD loci, and in their regulation (Figs 2, 3 and 7). The only other known example of non-TAD associated *flp* genes is one of the two *Agrobacterium tumefaciens flp* pili^45^.

The *B. bacteriovorus* TAD-encoding *flp* pilins (*flp1*, *flp2*) and *flp4* are expressed during AP in wild type cultures as well as in planktonic and in biofilm HI cultures (possibly in the AP cells of these unsynchronized cultures). Flp1 (Bd0119), along with other TAD locus proteins Bd0110, Bd0111, Bd0112, Bd0113, was also detected by proteome analyses, but Flp2 and Flp4 were not detected, possibly because their hydrophobicity and small mass make detection of Flp pilins very difficult^33^. *flp3* was silent under all various conditions we tested and as such is the first documented case of a *flp* pilin gene in gram-negative bacteria that was not found to be expressed. *flp*3 does not appear to be a pseudogene and it may be expressed under other environmental conditions.

While a type IVa pilus gene had been shown to be essential for *B. bacteriovorus* predation^14, 17^, no functional analysis of type IVb pilin genes have been performed in *B. bacteriovorus* ^33^. Since type IVb pilins are crucial for physical interactions with the eukaryotic host cell in pathogenic bacteria^48–50^ we posited they might also play an important role in BALO predation.

Attempts at deleting *flp1*, *flp2* and *flp4* in a wild type background were unsuccessful. In contrast, a *flp3* wild type knockout was viable, in accordance with its lack of expression in both AP and GP. In an HI background, triple *flp1-flp2-flp4*, double *flp1-flp2*, as well as all individual *flp* deletions yielded viable strains that could be grown axenically on a rich medium but were unable to prey. The *flp* genes were not interchangeable as their mutants were not complemented by each other or by the *flp3* gene. Thus, each of *flp1, flp2* and *flp4* is required for predation, and each  may have a specific role but none is crucial to the cell cycle, as mutants in an HI background were viable.

*B. bacteriovorus*, and all other BALO, include both type IVa and type IVb pilin genes. These pilin-encoding genes differ in their sequence characteristics and in the systems that assemble, process and export them^35^, and different functions have been attributed to each. While in most bacteria PilA pilins are responsible for surface-dependent motility and attachment, the Flp pilins were found to be involved only in surface attachment^38–44^. In some pathogenic bacteria (such as *Pseudomonas aeruginosa*, *Aggregatibacter actinomycetemcomitans, Haemophilus ducreyi* and *Agrobacterium tumefaciens*) disturbance of *flp* and TAD components attenuated virulence as well as decreased non-specific surface adhesion that is required for the development of biofilms^38, 45, 51, 52^. In other pathogens, *flp* deletion reduced virulence or microcolony formation without affecting biofilm formation^53^, or bacteria-cell host interaction^49^. In *B. bacteriovorus*, deletion of the three active *flp* genes had no effect on attachment/adhesion and HI biofilm formation. It is remarkable that both *Bdellovibrio* type IVa and the type IVb pilin –encoding genes, while essential for predation, are unnecessary for adhering or binding to prey cells, suggesting that other adhesion proteins expressed in the AP may be responsible for that step (58).

We provide the first experimental evidence that expression of the TAD-associated *flp1* and *flp2* gene is regulated by FliA, a flagellar sigma factor, both in the periplasmic predator, *B. bacteriovorus* HD100, and in the epibiotic predator, *B. exovorus* JSS. Further analyses of promoter sequences suggested that TAD associated *flp* genes are also regulated by FliA in other BALOs. Notably, *flp4*, while co-expressed with the TAD-encoded *flp1* and *flp2* is not similarly regulated by FliA. In many bacteria FliA regulates flagellar and chemotaxis-related genes^46, 54^. In several pathogens, FliA has expanded to directly control virulence related genes, such as a cytotoxic protein from *Campylobacter jejuni*, or indirectly through cylic-di-GMP modulation as with the *E. coli* fimbriae or *Vibrio cholerae* cytotoxin^55–57^. These FliA-regulated traits are unlinked to the cell cycle. In contrast, we previously found that in *B. bacteriovorus* FliA expanded to become the “AP master regulator” controlling about 77% of the AP genes^23^. Accordingly, in *B. bacteriovorus* FliA has turned into an essential gene: *B. bacteriovorus* FliA knockout was lethal both in wild type and HI mutants, indicating that FliA has expanded to regulate vital functions, a situation not known in any other bacterium. FliA regulation thus appears to be an indispensable component of obligate predators and a central regulator of the *Bdellovibrio* cell cycle.

Although functional analyses show that type IVa and type IVb pilins mutants are phenotypically undistinguishable from each other, genetic and biochemical analyses suggest that  each may have a different role in signaling prey cells to the predator. We propose to integrate our data into previously proposed models^8, 18, 20^. BALOs reversely bind the prey outer membrane (with a yet unknown component), followed by irreversible anchoring to a prey envelope-associated structure^8^. Type IV pili with their varied pilins, bring about the sensing, transduction, and an initial output of the interaction in the form of tightening of the predator-prey complex. According to a recent study, transition from AP to GP defines a third phase in *B. bacteriovorus*’ life cycle – the transition phase (TP)^8^. The TP expresses a specific transcription profile in response to signals triggered by the prey. Characteristically, response to the prey envelope results in *bd0108* shutdown, and in *pilA* and *fliA* down-regulation as the first step resulting from attachment to the prey. We suggest that type IVa and type IVb pili generate differentiated signaling outputs: type IVa pilus sensing leads to its retraction through Bd0108–Bd0109^18^ (AP genes not regulated by FliA) initiating a signal sensed at the invasive pole of the predatory cell^20^. This signal could be further processed through cyclic-di-GMP regulation^20^, leading to *bd0108* and other non-*fliA* regulated genes (*pilA*) downregulation; the signal generated by interacting type IVb pilins during attachment and further, during penetration and establishment in the periplasm, brings about alterations in *Bdellovibrio* *FliA-*regulated genes, including *flp* locus genes and pilin genes. This two-pronged signaling scheme, which still has to be validated, would ensure a rapid response based on enzymatic activation (c-di-GMP signaling) followed by a somewhat slower change at the transcription level.

## Experimental Procedures

### Bacterial strains and growth conditions

*B. bacteriovorus* HD100 wild type (Table S4) was grown with 10^9^ cells.ml^−1^ *E. coli* prey cells in diluted nutrient broth (DNB) at pH 7.4, supplemented with 2 mM CaCl_2_ and 3 mM MgCl_2_^58^. HI strains were grown in peptone-yeast extract medium (PYE) similarly supplemented with CaCl_2_ and MgCl_2_^58^. Unless mentioned otherwise, kanamycin and streptomycin were added to final concentrations of 25 µg.ml^−1^ and 100 µg.ml^−1^, respectively.

### Predation assay of the mutated and complemented strains

The tested strains were grown axenically to OD_600_ 0.7, centrifuged, washed twice in DNB, and suspended in 5 ml of OD_600_ 1 of *E. coli* in DNB supplemented with the appropriate antibiotic. *E. coli* were counted by serial dilution on LB agar every 24 hours for 120 h. Plates were incubated overnight at 28 °C.

### Orthologs identification and synteny analysis

Genuine orthologs were identified using the BLAST Score Ratio (BSR) approach^59^ using a ratio cutoff of 0.2. BSR similarity measure is the ratio between the blast score of one protein to another with the blast score of the protein to itself.

### *flp* pilins identification

A position weight matrix (PWM) was generated with the MEME program^60^ using *flp* pilins sequences from the Pfam database (entry PF04964)^61^, Fig. 3. The PWM was searched against selected BALO genomes using the MAST program^61^. Only high scoring hits (expected values < 0.1) within short (<100 amino acids) proteins or ORFs were further considered.

### Biofilm formation assay

*B. bacterivorous* HI M11.1 was grown from an initial 100 μl OD_600_ 0.7 in 96 well plates at 28 °C. Biofilm formation was measured every 24 hours using the standard crystal violet staining procedure^13^.

### Sample collection and RNA extraction

RNA was extracted using a MasterPure kit (epicenter) according to the manufacturer’s instructions. This was followed by an additional DNAse treatment using TurboDNASE kit (ambion). RNA integrity was validated by agarose gel electrophoresis. All the RNA samples were tested for DNA contamination by PCR using 16 S rRNA gene primers (8 f and 1492 R in Table S5). RNA extraction from biofilms was performed in glass test tubes: a turbid culture of the host independent strain M11.1 was diluted to OD_600_ 0.7 into 1 ml fresh PYE for 4 days. Next, the planktonic phase (AP) cells were harvested for RNA extraction. The biofilm was washed three times with HEPES buffer then immersed in RNALatter (Ambion), extracted from the test tube by scraping the flanks, centrifuged and kept at −80 °C until RNA was extracted. Samples were collected as described previously^8^: 1 ml of each time point was centrifuged and immersed in RNAlatter and then kept in −80 °C until RNA was extracted.

### Semi-quantitive RT-PCR (sqRTPCR)

100 to 200 ng of RNA were used for cDNA synthesis using Improm II kit (Promega) according to the manufacturer’s protocol. 50 µl of RNAse free water were added to the cDNA mixture to a final volume of 80 µl. sqRTPCR was performed using 1 μl of a diluted cDNA mixture, primers as listed in Table S5 and a Readymix polymerase (Lambda biotech) according to manufacturer’s instructions. Gels were recorded using a DNR MiniBIsPro BioImager with the DNR Gel Capture software with no further processing (DNR Bio-Imaging Systems, Israel).

### Proteome analyses

Analysis of the HD100 proteome was performed by MS. SDS-PAGE separation and digestion was performed as previously described^62^. The resulting peptide mixtures were fractioned by with strong cation exchange and analyzed by subsequent high-performance liquid chromatography coupled to an Orbitrap XL. MS data (survey and fragmentations scans) was analyzed using Thermo Proteome Discoverer™ (Thermo Fisher Scientific, Version 1.4.1.12) through SEQUEST HT search engine with a UniProt database (5^th^ November, 2015) containing the whole HD100 proteome^63^. Proteins were considered, if they were identified with at least two unique peptides and a minimum score of 20.

### Plasmid construction

All plasmids were constructed by the “Restriction Free” (RF) method as described by^64^. PCR was performed using Q5 polymerase (New England Biolabs) according to manufacturer instructions. Plasmids backbones and primers are listed in Tables S4 and S5, respectively.

For the purpose of in-frame deletions, 500–1000 bp long DNA fragments were amplified from both sides of the target gene and fused together by the RF method to the suicide plasmid pSSK10^65^. Knockout primers were designed to leave a 16–17 amino acid remaining to avoid upstream polar effects.

Complementation was carried by pPROBE-NT, which carried the examined gene with its promoter (usually 200–400 bp upstream to the translation start site).

Examined *fliA* orthologs were cloned to replace the gIII secretion signal peptide, downstream to the arabinose-induced promoter in pBAD/gIII (Invitrogen). Examined promoters were cloned upstream to a promoter-less full-length β-galactosidase (lacZ) in pBBR1-MCS2-LacZ^66^.

### Conjugation in *B. bacteriovorus*

*E. coli* donor strains were grown with the appropriate antibiotics (listed in Table S4) to an OD_600_ of 0.4, then washed twice with DNB and concentrated 10-fold. A 100 µl volume of each donor was mixed with 100 µl of *B. bacteriovorus* HD100 or *B. bacteriovorus* M11.1 from a turbid culture or with an *E. coli*-*B. bacteriovorus* HD100 overnight- dual culture. The mixture was then spread over a sterile membrane that was placed upon a DNB agar plate. Following overnight incubation at 28 °C, the membrane was washed with 1 ml of DNB, diluted and plated on *E. coli* double layer agar or PYE agar plates supplemented with 50 µg.ml^−1^ streptomycin and 25 µg.ml^−1^ kanamycin. When *E. coli* MFDpir was used, diaminopimelic acid (DAP) was added to a final concentration of 100 µg.ml^−1^.

### *B. bacteriovorus* knockouts

After conjugation with the appropriate pSSK10 derived construct, plaques or colonies were picked up and checked for merodiploidicity by PCR (Table S4). Selected plaques or colony were transferred into 5 ml of OD_600_ 1 of an *E. coli* suspension or to PYE, respectively, supplemented with streptomycin and grown for several days. An aliquot was transferred to OD_600_ 1 of an *E. coli* suspension or PYE both supplemented with streptomycin and 5% sucrose and grown for 24 and 72 hours, respectively, then diluted and plated on *E. coli* double layer agar or PYE agar plate both supplemented with 50 µg.ml^−1^ streptomycin. Plaques and colonies appeared after a week of incubation. Plaques were transferred to a 96 wells plate, each well containing 150 to 200 μl of a OD_600_ 1 *E. coli* suspension. HI colonies were transferred to a grid PYE agar plate (53 colonies per plate), then after appropriate incubation, samples were examined for allelic exchange by PCR. Parental strains (WT HD100 or HI) and the appropriate merodiploid strain were used in each PCR reaction as a control.

Selection for *fliA* knockout strain was performed with the following modifications: Recombinants were screened as above in counter-selection tubes containing PYE and 5% sucrose. After 2, 4 and 5 days (instead of after 3 days in the standard procedure), suspensions were plated on PYE agar and incubated for a week. Colonies were examined for allelic exchange by PCR. Thereafter, plates were incubated for an additional 2 to 3 weeks under humid conditions, to enable the development of slowly growing colonies, typically 10 to 20 which were examined for allelic exchange by PCR. This experiment was performed three times.

### *E. coli* fliA knockout

*E. coli fliA* was knocked out using a Lambda-red recombination system^67^. Briefly, *E. coli* DH5α/pKD46 was induced for lambda red recombinase by adding 0.05% arabinose. A linear DNA fragment containing a chloramphenicol resistance cassette flanking 30 bp with the corresponding upstream and downstream to *E. coli fliA* was created by PCR using primers: fliAlambdared-F and fliAlambdared-R and pKD3 as a template. Next, the liner fragment was electroporated into the induced lambda red *E. coli* DH5α/pKD46. This resulted in chloramphenicol resistant *E. coli* colonies that were isolated and validated for *fliA* knockout using primers Colifliadel-R and Colifliadel-F.

### *E. coli* - *Bdellovibrio* attachment assay

Attachment assays were performed according to Milner *et al*.^20^ with slight modifications. HI and HI *flp* mutants were grown in PYE medium to an OD_600_ of 0.5, washed twice in DNB, and mixed with *E. coli*. The experiments were carried out at the final cell concentrations of 10^6^ and of 10^7^ ml^−1^. The *Bdellovibrio - E. coli* co-culture was incubated for recovery of 30 min in 28 °C, then concentrated by low speed centrifugation (4000 rpm for 10 min), the pellet was re-suspended in fresh DNB and re-incubated in 28 °C. 10 µl were taken at different time points, placed on positively charged polylysine-coated adhesive microscope slide (X-tra slides, Leica) and examined under phase contrast microscopy. As a negative control, *E. coli* was replaced with *Bacillus subtilis*, a gram-positive bacterium that *Bdellovibrio* does not attach to.

### Examination of *B. bacteriovorus and B. exovorus flp* pilin gene promoters response to FliA in an *E. coli* heterologous system

Examined promoters were cloned upstream to β-Galactosidase (lacZ) in pBBR1-MCS2-LacZ. The resulting constructs were introduced into *E. coli* DH5α ∆*fliA* together with FliA cloned under arabinose-induced promoter in pBAD. The endogenous *E. coli fliA* was knocked out in order to reduce the background signal. Each *E. coli* strain carrying the two plasmids was grown overnight, then diluted into fresh LB to OD_600_ 0.05, supplemented with either 0.5% glucose or 0.5% arabinose. When cultures reached mid-log (OD_600_ 0.4–0.6), bacteria were harvested and measured for LacZ activity following a standard protocol^68^. Final β-Galactosidase activity was calculated by the following formula:

$${\rm{Final}}\,\mathrm{activity}\{\mathrm{Miller}\,\mathrm{units}\}:({{\rm{Induced}}}^{{\rm{arabinose}}}{\rm{bd}}3318)-({{\rm{repressed}}}^{{\rm{glucose}}}{\rm{bd}}3318).$$

In this manner any background signals stemming from *E. coli* were eliminated.

## Electronic supplementary material

Supplementary material
