# Supplementary material for: Identification and Characterization of Differentially-Regulated Type IVb Pilin Genes Necessary for Predation in Obligate Bacterial Predators
Source: Sci Rep. 2017 Apr 21;7:1013. doi: 10.1038/s41598-017-00951-w (PMC5430801; doi:10.1038/s41598-017-00951-w)
Supplement: Supplementary file 1 — Supplementary material [file 41598_2017_951_MOESM1_ESM.pdf]

Identification and Characterization of Differentially-Regulated Type IVb Pilin Genes Necessary for Predation in Obligate Bacterial Predators

Ofir Avidan[a], Margarita Petrenko[b], René Becker[c], Sebastian Beck[c], Michael Linscheid[c], Shmuel Pietrokovski*[a], and Edouard Jurkevitch*[b].

Department of Molecular Genetics, The Weizmann Institute of Science, Rehovot, Israel[a], Department of Plant Pathology and Microbiology, Faculty of Agriculture, Food and Environment, The Hebrew University of Jerusalem, Rehovot, Israel[b], Department of Chemistry, Humboldt-Universität zu Berlin, Berlin, Germany[c].

Running head: Type IVb pili in bacterial predators

*Corresponding Authors: Edouard Jurkevitch, edouard.jurkevitch@mail.huji.ac.il and Shmuel.Pietrokovski@weizmann.ac.il

Supplementary data

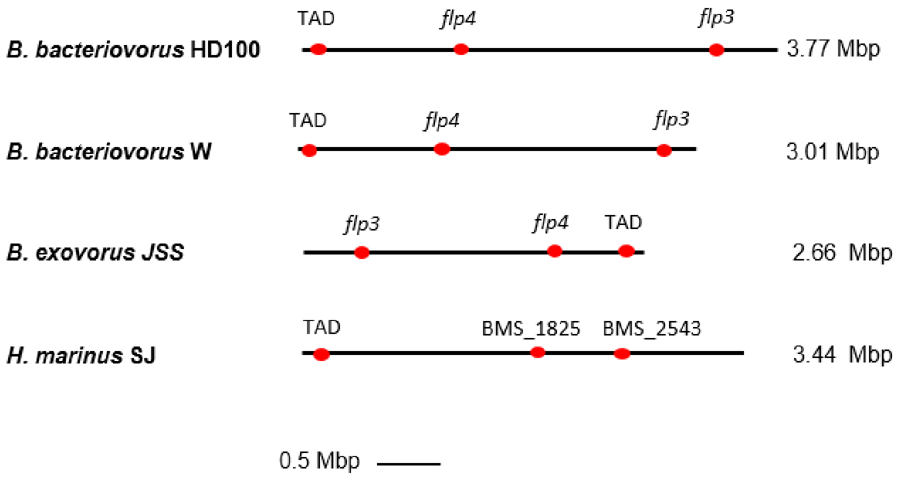

Figure S1. Schematic positions of *flp* genes on fully assembled BALO genomes. Genome sizes are shown on the right side of each line, with the name of the organism on the left.

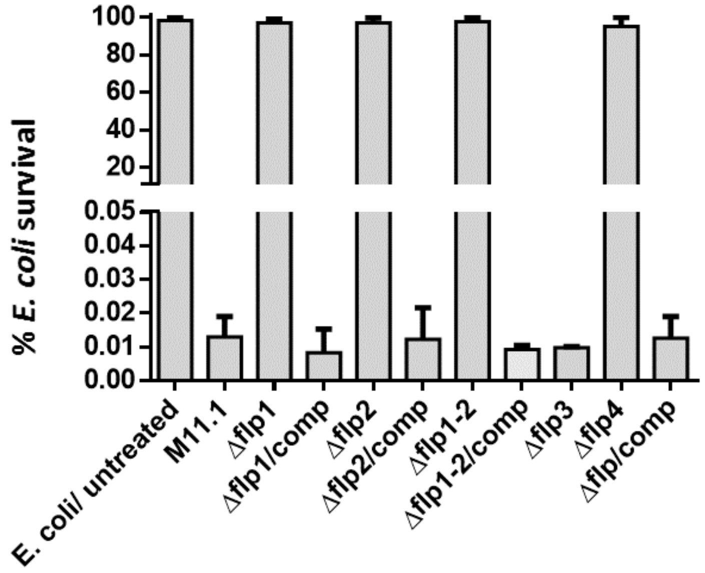

Figure S2. Predatory dynamics of *B. bacteriovorus* HI M1.11, of the various *flp* mutants, and of their complemented strains. Predator and prey (*E. coli*) were counted by plating serial dilutions. Data shown in figure 5 are derived from the 96h time point. The data show counts obtained after 96h of co-incubation.

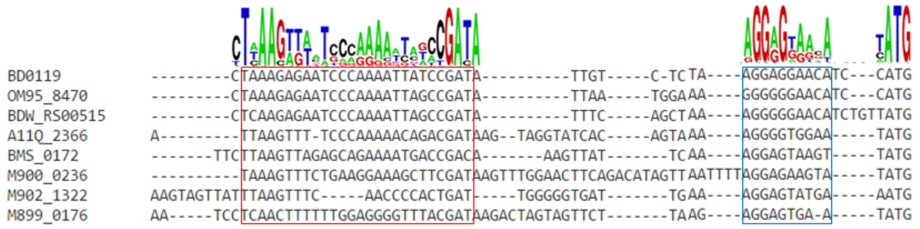

Figure S3. Promoter analysis of *flp1* from different BALOs

Promoter comparison of TAD loci in different BALOs reveals a putative FliA recognition site (marked in red box) 22-26 nucleotides upstream to the ATG start codon [1, 2]. A conserved ribosome binding site is found 3-9 nucleotides upstream to the ATG start codon (marked in blue box) [3]. Multiple alignment was performed using MAFFT [4], nucleotide logos were created by WebLogo [5].

Table S1. **Comparison of syntenous regions in AP core genes between different BALOs.** Eleven of the 43 AP core genes are in four different loci (grey shading). These syntenous loci are shared among all examined BALOs (four *Bdellovibrio*, three *Bacteriovorax* and one *Halobacteriovorax* strains).

		Product	*Bdellovibrio bacteriovorus* HD1	*Bdellovibrio* st W		*Bdellovibrio* st. ArHS		*Bdellovibrio exovorus* JSS		*Halobacteriovorax mar*		*Bacteriovorax* sp. BAL6_X		*Bacterivorax* sp. BSW11_IV		*Bacteriovorax sp. Seq25_V*	
**FliA regulation***	**AP vs. GP induction**		**locus**	**tag locus**	**BSR**	**tag locus**	**BSR**	**Tag locus**	**BSR**	**Tag locus**	**BSR**	**Tag locus**	**BSR**	**Tag locus**	**BSR**	**Tag locus**	**BSR**
Unkown	6.251572327	cell wall-associated protein precursor	Bd0109	BDW_RS00470	0.874781	OM95_08515	0.936077058	A11q_2375	0.629597198	BMS_0182	0.267075306	M902_1312	0.26619965	M899_018	0.252189142	M900_0246	0.254816112
yes	22.36470588	hypothetical protein	Bd0110	BDW_RS00475	0.85	OM95_08510	0.898275862	A11q_2374	0.594827586	BMS_0181	0.351724138	M902_1313	0.336206897	M899_018	0.327586207	M900_0245	0.289655172
yes	64.225	Flp pilus assembly protein TadA	Bd0111	BDW_RS00480	0.872836	OM95_08505	0.899467377	A11q_2373	0.671105193	BMS_0180	0.416111851	M902_1314	0.400133156	M899_018	0.421438083	M900_0244	0.427430093
yes	456.2	putative flp pilus assembly protein Cp	Bd0113	BDW_RS00490	0.85173	OM95_08495	0.896210873	A11q_2371	0.499176277	BMS_0178	0.261943987	M902_1316	0.23723229	M899_018	0.266886326	M900_0242	0.257001647
yes	330.2153846	hypothetical protein	Bd0119	BDW_RS00515	0.550413	OM95_08470	0.582644628	A11q_2366	0.321487603	BMS_0172	0.309090909	M902_1322	0.289256198	M899_017	0.333884298	M900_0236	0.318181818
Unkown	6.962987013	nitrogen fixation specific regulatory pr	Bd0156	BDW_RS13470	0.914312	OM95_11095	0.943482224	A11q_0154	0.775752051	BMS_3271	0.464904284	M902_1682	0.474020055	M899_335	0.474020055	M900_2669	0.484958979
no	40.48796296	serine protein kinase	Bd0228	BDW_RS00825	0.947368	OM95_08080	0.962807018	A11q_0408	0.863157895	BMS_1500	0.678596491	M902_2929	0.682105263	M899_192	0.689824561	M900_1039	0.690526316
no	81.85744681	hypothetical protein	Bd0229	BDW_RS00830	0.881333	OM95_08075	0.858666667	A11q_0409	0.796	BMS_1501	0.554666667	M902_2930	0.556	M899_192	0.572	M900_1040	0.581333333
no	73.98888889	putative sporulation protein R	Bd0231	BDW_RS00835	0.889302	OM95_08070	0.933953488	A11q_0410	0.765581395	BMS_1502	0.503255814	M902_2931	0.502325581	M899_192	0.52	M900_1041	0.505116279
no	9.995	acetyltransferase	Bd0335	BDW_RS01210	0.488889	OM95_06105	0.66031746	A11q_2185	0.419047619	BMS_1252	0.368253968	M902_1227	0.365079365	M899_220	0.374603175	M900_1674	0.419047619
Unkown	14.39360465	RNA-binding protein	Bd0339	BDW_RS01230	0.604348	OM95_06085	0.713043478	A11q_1141	0.52173913	BMS_1132	0.241304348	M902_0787	0.281304348	M899_064	0.47826087	M900_1021	0.469565217
Unkown	4.721428571	maf protein	Bd0469	BDW_RS01640	0.680203	OM95_05610	0.736040609	A11q_0529	0.461928934	BMS_0695	0.291878173	M902_0656	0.276649746	M899_112	0.281725888	M900_0803	0.284263959
yes	95.05	Flp pilus assembly protein TadC	Bd0470	BDW_RS01645	0.874517	OM95_05605	0.903474903	A11q_0530	0.700772201	BMS_3229	0.305019305	M902_1706	0.322393822	M899_331	0.333976834	M900_2630	0.337837838
yes	173.8	putative secreted protein	Bd0471	BDW_RS01650	0.714286	OM95_05600	0.773109244	A11q_0531	0.521008403	BMS_3228	0.385294118	M902_1707	0.262184874	M899_331	0.36092437	M900_2629	0.378571429
Unkown	11.39285714	two-component response regulator	Bd0569	BDW_RS03475	0.209761	OM95_01150	0.785249458	A11q_0702	0.442516269	BMS_1338	0.284164859	M902_2587	0.279826464	M899_042	0.260303688	M900_1317	0.268980477
Unkown	1636.066667	flagellin	Bd0604	BDW_RS02065	0.769373	OM95_01300	0.778597786	A11q_2087	0.57195572	BMS_0169	0.5	M902_1325	0.533210332	M899_017	0.507380074	M900_0156	0.509225092
yes	50.009375	flagellin	Bd0606	BDW_RS02070	0.888278	OM95_01310	0.912087912	A11q_1936	0.637362637	BMS_0169	0.575091575	M902_1325	0.595238095	M899_033	0.586080586	M900_1164	0.58974359
yes	7.206363636	hypothetical protein	Bd0740	BDW_RS02520	0.894133	OM95_01910	0.911989796	A11q_0683	0.698979592	BMS_1302	0.279336735	M902_1192	0.304846939	M899_047	0.317602041	M900_1724	0.293367347
yes	44.38666667	cpaF protein	Bd0793	BDW_RS02700	0.926471	OM95_02080	0.949197861	A11q_0709	0.755347594	BMS_1635	0.562834225	M902_3126	0.540106952	M899_198	0.557486631	M900_A0343	0.546791444
Unkown	14.27857143	protease	Bd1077	BDW_RS03730	0.776971	OM95_03625	0.860995851	A11q_2572	0.211618257	BMS_1295	0.270746888	M902_1183	0.268672199	M899_048	0.25	M900_1731	0.270746888
yes	242.9	Serine protease/subtilase	Bd1283	BDW_RS04355	0.80333	OM95_04055	0.833506764	A11q_1642	0.614984391	BMS_1331	0.238293444	M902_2591	0.260145682	M899_043	0.237252862	M900_1328	0.25182102
Unkown	9.901204819	fimbrial protein pilA	Bd1290	BDW_RS04385	0.486979	OM95_04025	0.442708333	A11q_1638	0.278645833	BMS_0122	0.251822917	M902_1364	0.22265625	M899_012	0.244791667	M900_0191	0.240625
Unkown	6.137037037	hypothetical protein	Bd1303	BDW_RS04415	0.564202	OM95_03985	0.498054475	A11q_0269	0.544747082	BMS_0889	0.463035019	M902_2876	0.36848249	M899_041	0.412451362	M900_1288	0.350583658
Unkown	5.709090909	hypothetical protein	Bd1304	BDW_RS04410	0.527211	OM95_03980	0.540816327	A11q_0270	0.540816327	BMS_0890	0.421768707	M902_2877	0.380952381	M899_041	0.414965986	M900_1290	0.338095238
yes	8.884	adventurous gliding motility protein R	Bd1481	BDW_RS04965	0.904564	OM95_12565	0.937759336	A11q_0732	0.200622407	BMS_1382	0.27593361	M902_0277	0.228215768	M899_039	0.29253112	M900_0340	0.263485477
no	11.25327869	hypothetical protein	Bd1710	BDW_RS06010	0.794207	OM95_00380	0.853658537	A11q_1453	0.679878049	BMS_3011	0.263719512	M902_1877	0.295731707	M899_311	0.254573171	M900_0570	0.268292683
no	58.61724138	hypothetical protein	Bd1711	BDW_RS06015	0.725296	OM95_00385	0.843873518	A11q_0521	0.225296443	BMS_2744	0.31027668	M902_2071	0.227272727	M899_277	0.260869565	M900_2114	0.272727273
yes	104.36	methyl accepting chemotaxis protein	Bd1872	BDW_RS14185	0.556064	OM95_11750	0.652173913	A11q_1203	0.315789474	BMS_2634	0.267734554	M902_0335	0.260869565	M899_277	0.274599542	M900_0660	0.297482838
yes	154.75	NtrC family transcriptional regulator	Bd1891	BDW_RS13470	0.230911	OM95_11095	0.229070837	A11q_0933	0.220791168	BMS_3271	0.217111316	M902_1682	0.217111316	M899_335	0.226310948	M900_2669	0.218951242
Unkown	5.482926829	copper-transporting ATPase copA	Bd2224	BDW_RS10145	0.351738	OM95_02570	0.709611452	A11q_1716	0.338786639	BMS_0951	0.33060668	M902_0916	0.340149966	M899_085	0.319700068	M900_2024	0.309475119
Unkown	5.766666667	cation efflux system protein, AcrB/Acr	Bd2226	BDW_RS10560	0.564191	OM95_00630	0.63698967	A11q_0509	0.621249385	BMS_1001	0.2538121	M902_2596	0.255779636	M899_096	0.243482538	M900_1835	0.247417609
yes	69.16923077	integral HD domain-containing protei	Bd2421	BDW_RS08820	0.789357	OM95_10535	0.829268293	A11q_0992	0.649667406	BMS_3015	0.392461197	M902_1873	0.392461197	M899_312	0.381374723	M900_0566	0.383592018
yes	272.2	uridine kinase	Bd2645	BDW_RS09380	0.71327	OM95_00950	0.746445498	A11q_1748	0.646919431	BMS_0599	0.549763033	M902_1405	0.279620853	M899_008	0.312796209	M900_1465	0.504739336
Unkown	6.538888889	ABC transporter ATP-binding protein	Bd2748	BDW_RS07260	0.312044	OM95_09175	0.76459854	A11q_2447	0.306569343	BMS_2387	0.211678832	M902_0485	0.21350365	M899_045	0.253649635	M900_1313	0.321167883
Unkown	4.598717949	flagellin	Bd3052	BDW_RS11010	0.957486	OM95_10055	0.972273567	A11q_1936	0.71349353	BMS_0169	0.567467652	M902_1325	0.578558226	M899_017	0.563770795	M900_0156	0.554528651
Unkown	5.393103448	ABC-type multidrug transporter, ATPa	Bd3098	BDW_RS11190	0.772688	OM95_02870	0.823681936	A11q_1580	0.240276577	BMS_1922	0.254105445	M902_2328	0.229040622	M899_189	0.273984443	M900_A0168	0.247191011
yes	41.86666667	hypothetical protein	Bd3148	BDW_RS11375	0.741815	OM95_05390	0.835565476	A11q_1998	0.441220238	BMS_2759	0.235863095	M902_1990	0.244047619	M899_279	0.202380952	M900_2131	0.24702381
no	9.429268293	RNA polymerase sigma factor for flage	Bd3318	BDW_RS11975	0.95619	OM95_04705	0.986666667	A11q_0617	0.76	BMS_0566	0.577142857	M902_0431	0.603809524	M899_297	0.59047619	M900_0686	0.6
Unkown	15.81666667	protein-glutamate methylesterase	Bd3467	BDW_RS12630	0.799722	OM95_01190	0.242002782	A11q_1211	0.331015299	BMS_2632	0.314325452	M902_0336	0.326842837	M899_262	0.343532684	M900_2470	0.321279555
Unkown	12.77912088	penicillin-binding protein transpeptida	Bd3522	BDW_RS12790	0.663774	OM95_07780	0.812364425	A11q_2242	0.489154013	BMS_0103	0.221258134	M902_1381	0.227765727	M899_011	0.252711497	M900_0174	0.247288503
Unkown	5.319141104	DNA polymerase III gamma and tau su	Bd3731	BDW_RS13285	0.711599	OM95_11340	0.780564263	A11q_0202	0.47492163	BMS_0050	0.228056426	M902_1428	0.214733542	M899_006	0.221003135	M900_0126	0.219435737
yes	9.694202899	ATP-dependent protease ATP-binding	Bd3753	BDW_RS13360	0.915332	OM95_11215	0.969107551	A11q_0178	0.803203661	BMS_2640	0.632723112	M902_2205	0.638443936	M899_264	0.627002288	M900_2487	0.627002288
yes	16.91875	ATP-dependent Clp protease proteoly	Bd3754	BDW_RS13365	0.863326	OM95_11210	0.881548975	A11q_0179	0.822323462	BMS_2641	0.569476082	M902_2204	0.555808656	M899_264	0.576309795	M900_2488	0.562642369

Table S2. *flp3* and *flp4* synteny in diverse *Bdellovibrio* species.
Orthologs of *B. bacteriovorus* HD100 *flp3* and *flp4* genes and their flanking genes were identified in other *Bdellovibrio* strains using sequence similarity. BSR values are indicated beneath the gene names.

Bdellovibrio bacteriovorus HD100	bd3296	Bd3297(flp3)	bd3298	bd2719	Bd4000 (flp4)	bd2720
Bdellovibrio sp. ArHs	OM95_4800 (0.7)	OM95_4795 (0.6)	OM95_4790 (0.2)	OM95_9275 (0.86)	OM95_9270 (0.7)	OM95_9260 (0)
Bdellovibrio bacteriovorus W	RDW_RS09640 (0.80)	RDW_RS09645 (0.58)	RDW_RS09650 (0.65)	RDW_RS11875 (0.62)	RDW_RS11880 (0.71)	RDW_RS11885 (0.28)
Bdellovibrio exovorus JSS	A11Q_631 (0.66)	A11Q_630 (0.4)	A11Q_299 (0)	A11Q_1810 (0.71)	A11Q_1811 (0.56)	A11Q_1812 (0.25)

Table S3. Proteins identified with mass spectrometry. The peptide sequences marked in yellow were detected. Identification was performed with Proteome Discoverer™ (Thermo Fisher Scientific, Version 1.4.1.12).

Bd0110	hypothetical protein				Sequence coverage: 43.30 %	
10	20	30	40	50	60	70
MSFLFNEWVM	IPLFGICVFV	IVILWADKAI	AWLHKRSLGQ	RDEVIKILRV	MGMDVDEKKV	TLIIILLMSFG
80	90	100	110	120	130	140
LGALVFLIFW	PSVLMGAFFG	ASITVAGWQL	PLLLVRMIYE	QRCTKFVDQM	VDGLTIMANG	IKAGSNPQES
150	160	170	180	190	200	210
MKRVVEIMGN	PMSQEFAQVL	YQMQVGDSFE	SALNDLGNRI	PRPDVQMFVT	SINILKETGG	NLAETFQTIV
220	230	240	250	260	270	280
LVVRERQKVE	KKIQALTAQG	MMQGIIVTLI	PFILMAVFLV	IDPAFIKPMF	NTTLGLVLLA	AMLGLQIIGG
290						
VLIKKLVTIK	V					

Bd0111	Flp pilus assembly protein TadA				Sequence coverage: 85.52 %	
10	20	30	40	50	60	70
MAINPNCNLI	AVVGGKGGVG	KSVFAANFAC	TIMNELRSQV	LLIDADARSV	GDQNVIMGLK	PQKTLKELAS
80	90	100	110	120	130	140
FQGSLNSQPM	NTLVTMHQSG	LAYLGAVRGP	EESLNINPDL	LGKLVEFFSR	AYKFIIVDVG	TELGPAQMAV
150	160	170	180	190	200	210
LQEATAIMIV	TTPEVLVVTQ	TQRLVNELLS	ATLPKDMFQL	VINKASPTGL	SPQTISNQLQ	LPFLGIIPQD
220	230	240	250	260	270	280
EATSMMSLQK	YTPFVISAPK	APVTAAYYDV	ARKLTGGILQ	RLKTLSRPKP	APAAAAAAGD	SASTGSTQGM
290	300	310	320	330	340	350
DARTLLKIRI	HNELIRTVDL	KKLLVDGKQD	ENKEKEVREK	TKREITLIVD	REAPDVAREE	RSKLIKEVLE
360	370	380	390	400	410	420
EALGLGPLED	LLADPDVTEI	MVNGNKRVFV	EKSGKVQLSP	VTFTSNDHLR	RIIERIVTPL	GRQINDSTPY
430	440	450	460	470	480	490
VDARLKDGSR	VNAVIEPLAI	DGPALTIRKF	KKGGITAEKY	IGYGSITKNM	IDFLRICVEN	GLNVVISGGT
500	510	520	530	540	550	560
GSGKTSLLNM	LSSFIPSNER	VITVEDAAEL	QLQQEHVVRL	ETRPASMEGS	NAIHIRDLIK	NALRMRPDRI
570	580	590	600	610	620	630
IVGECRDGAA	LDMLQAMNTG	HDGSMTTTHA	NSPRECVARL	ETLCMMSGMD	LPMRAIREQI	AGAVNLIVQI
640	650	660	670	680	690	700
SRLSDGSRKI	LSITEVAGMQ	GDVVTLAEIF	RFKETGYDKN	RKIQGVFQAT	GTIPSFIQKL	SDKGVVIPRE
710	720	730				
IFANDPNAGT	PAAAAAAKPP	IPAAMAPKMP	GVAPVKKSG			

Bd0112	Putative pilus assembly transmembrane protein				Sequence coverage: 85.46 %	
10	20	30	40	50	60	70
MRRKILITGL	LSLFMISGSV	AWAQEELEAS	PTSSTEGEEG	VYRSRKFINL	TLGIEQDEKL	PPLPDSIEFK
80	90	100	110	120	130	140
GDFRRIVTAA	YAKDLNVIRF	TPRGEGFATL	TIHDKRNGKI	VAEFRIDVKK	SKLDKVVREM	RALLGDIEGI
150	160	170	180	190	200	210
NIKIVNNRVV	VDGQILLPKD	LARIYNVVQQ	FGEQASSLVT	LSPLAQKKIA	EFIARDINNP	EIEVRAVNEK
220	230	240	250	260	270	280
IILQGWANSD	EEAKRAEIIA	KTYLPDIVIE	AAEEKGVIKK	RRPANDGVIN	LIQIKEAPAK	PPSKMIQLVV
290	300	310	320	300	340	350
HYVELNKDYS	KAFKFQFTPE	LGDNSQMTFQ	TGGDSPGGVI	SSITGTVSNL	LPKLNWAKQH	GHARVLESTS
360	370	380	390	400	410	420
LIVEDGKKGE	IKQVTNQPYP	VIGKDGTQGT	AFAEVGIVTA	ITPVLLGEKS	GSVHMEMAFK	VSSLLGNTPS
430	440	450	460	470	480	490
GAPITSANEM	TSTVTVRDRQ	SAAVGGLIRN	STSTGYNRPA	GQKNPIISLY	ASKDFIKQQS	QFVVFVTPIV

500<br>KTSASSGAEQ IKKKFRLRD		
Bd0113	putative flp pilus assembly protein CpaB	Sequence coverage: 58.14 %
10 20 30 40 50 60 70<br>MGSNETRNLW LSIAAGVFAT FLLYSYSQEK KAEYDKRFGS TKRVVVAKED IAEMQTIYDT MVETKELPAD<br>80 90 100 110 120 130 140<br>FIQPDAITIP DEIIGNVAAV PIRKGQMVVK NNLLTPGPDT GISLQVAPSK RAVTIPVDEV RGVAKLIRPG<br>150 160 170 180 190 200 210<br>DRIDIYAAVD SGKGVNQRRE VFTMMNDVVV LATGVSVVNN IPRMFELDST GKNLTQIALT GDTKYTTITV<br>220 230 240 250 260 270 280<br>EATPKEAQDL FYILSTAPGN LFMALRNPSD RTIPPRMPSS TSDSVLGKPM VSVDAAPAIA MPPRPYVPPV<br>290 300<br>QQQRAAPPPA QRPRANGFQT L		
Bd0119	hypothetical protein	Sequence coverage: 59.68 %
10 20 30 40 50 60<br>MKKFKNFSKK LLKNESGQGA TEYILLLVVV VALVVIFKDR IKTAMEEKVG SLASDITGFS GN		

Table S4: Strains and plasmids used in this study.

Strain	Genotype/Description	Reference
Bdellovibrio bacteriovorus HD100	Streptomycin resistant.	[6]
Bdellovibrio bacteriovorus M11.1	Spontaneous HI mutation of *Bdellovibrio bacteriovorus* HD100, streptomycin resistant.	[6]
E. coli SM10	Mating strain<br>KmR, thi-1, thr, leu, tonA, lacY, supE, recA::RP4-2-Tc::Mu, pir.	[7]
E. coli MFDpir	Mating strain, Diaminopimelic acid auxotroph.<br>MG1655 RP4-2-Tc::{ΔMu1::aac(3)IV-ΔaphA-Δnic35-ΔMu2::zeo} ΔdapA::(erm-pir) ΔrecA	[8]
E. coli DH5α	F– Φ80*lac*ZΔM15 Δ(*lac*ZYA-*arg*F) U169 *rec*A1 *end*A1 *hsd*R17 (rK–, mK+) *pho*A *sup*E44 λ– *thi*-1*gyr*A96 *rel*A1	
E. coli DH5α ΔfliA::Cam	E. coli FliA inactivated by chloramphenicol resistance cassette	This study
B. bacteriovorus M11.1/pPROBE-NT	Empty pPROBE-NT vector that was introduced M11.1	This study
B. bacteriovorus M11.1 Δ*flp1*-2/pPROBE-NT	Empty pPROBE-NT vector that was introduced to *flp1-2* knockout	This study
B. bacteriovorus M11.1 Δ*flp1*-2/pPROBE-NT ::*flp1*-2	Complement strain. pPROBE-NT ::*flp1*-2, that was introduced to *flp1-2* knockout	This study
B. bacteriovorus M11.1 Δ*flp2*/pPROBE-NT	Empty pPROBE-NT vector that was introduced to *flp2* knockout	This study
B. bacteriovorus M11.1 Δ*flp2*/pPROBE-NT:: *flp2*	Complement strain. pPROBE-NT ::*flp2* that was introduced to *flp2* knockout	This study
B. bacteriovorus M11.1 Δ*flp1*/pPROBE-NT	Empty pPROBE-NT vector that was introduced to *flp1* knockout	This study
B. bacteriovorus M11.1 Δ*flp1*/pPROBE-NT:: *flp1*	Complement strain. pPROBE-NT ::*flp1*, that was introduced to *flp1* knockout	This study
B. bacteriovorus M11.1 Δ*flp3*/pPROBE	Empty pPROBE-NT vector that was introduced to *flp3* knockout	This study

B. bacteriovorus M11.1 Δ*flp4*/pPROBE	Empty pPROBE-NT vector that was introduced to *flp4* knockout	This study
B. bacteriovorus M11.1 Δ*flp4*/pPROBE*::flp4*	Complement strain. pPROBE-NT ::*flp4* that was introduced to *flp4* knockout	This study
B. bacteriovorus M11.1 Δ*flp1*-2-4/pPROBE	Empty pPROBE-NT vector that was introduced to *flp1*-2-4 knockout. This strain was constructed by deleting *flp4* on the genetic background of M11.1 *flp1-2* knockout	This study
B. bacteriovorus M11.1 Δ*flp1*-2-4/pPROBE*:: flp1-2,flp4*	Complement strain. pPROBE-NT ::*flp1*-2,*flp4* was introduced to *flp1*-2-4 knockout strain	This study
E. coli DH5α ΔfliA::Cam/pBAD-bd3318, pBBR1MCS2-LacZ	*E. coli* ΔfliA that was co-transfected with promoterless lacZ and FliA ,bd3318, under arabinose induction	This study
E. coli DH5α ΔfliA::Cam/pBAD-bd3318, pBBR1MSC2 p*bd0119*::lacZ	*E. coli* ΔfliA that was co-transfected with lacZ under regulation of *B. bacteriovorus flp*1, bd0119 , promoter and FliA , bd3318, under arabinose induction	This study
E. coli DH5α ΔfliA::Cam/pBAD-bd3318, pBBR1MSC2 pbd0606::lacZ	*E. coli* ΔfliA that was co-transfected with lacZ under regulation of B. bacteriovorus flagellin, bd0606, promoter and FliA ,bd3318, under arabinose induction	This study
E. coli DH5α ΔfliA::Cam/pBAD-bd3318, pBBR1MSC2 p*bd32973*::lacZ	*E. coli* ΔfliA that was co-transfected with lacZ under regulation of *B. bacteriovorus flp*3 ,bd3297, promoter and FliA ,bd3318, under arabinose induction	This study
E. coli DH5α ΔfliA::Cam/pBAD-bd3318, pBBR1MSC2 p*bd4000*::lacZ	*E. coli* ΔfliA that was co-transfected with lacZ under regulation of *B. bacteriovorus flp4, bd4000,* promoter and and FliA ,bd3318, under arabinose induction	This study
E. coli DH5α ΔfliA::Cam/pBAD-a11q_617, pBBR1MCS2-LacZ	*E. coli* ΔfliA that was co-transfected with promoterless lacZ andB. Exovorus FliA ,A11q_617, under arabinose induction	This study
E. coli DH5α ΔfliA::Cam/pBAD-a11q_617, pBBR1MCS2 pa11q2366	*E. coli* ΔfliA that was co-transfected with lacZ under regulation of *B.exovorus flp*1, A11q_2366 , promoter and FliA, a11q_617, under arabinose induction	This study
E. coli DH5α ΔfliA::Cam/pBAD-a11q_617, pBBR1MCS2-LacZ *pA11q_1936*	*E. coli* ΔfliA that was co-transfected with lacZ under regulation of *B.exovorus* Flagellin, A11q_1936 , promoter and FliA, a11q_617, under arabinose induction	This study
E. coli DH5α ΔfliA::Cam/pBAD-a11q_617, pBBR1MCS2-LacZ *pA11q_630*	*E. coli* ΔfliA that was co-transfected with lacZ under regulation of *B.exovorus flp*3, A11q_630 , promoter and FliA, a11q_617, under arabinose induction	This study
E. coli DH5α ΔfliA::Cam/pBAD-a11q_617, pBBR1MCS2 pa11q_1811	*E. coli* ΔfliA that was co-transfected with lacZ under regulation of *B.exovorus flp*4, A11q_1811 , promoter and FliA, a11q_617, under arabinose induction	This study

Plasmids		
pBBR1MCS2-LacZ	pBBR based plasmid with promoter-less β-galactosidase (lacZ), ampicillin resistant	[9]
pPROBE-NT	Broad host range vector (pBBR ori), kanamycin resistant,	[10]
pBAD/gIIIA		Invitrogen
pSSK10	Suicide vector, R6K ori, SacB (Sucrose sensitive)	[11]
pKD46	Red recombinase expression plasmid, temperature sensitive.	[12]
pKD3	Template for Chloramphenicol cassette for gene inactivation	[12]
pBAD-bd3318	Expression of *B. bacteriovorus* FliA, bd3318, under arabinose inducible promoter. in pBAD/gIIIA, constructed using primers Bd3318-pBAD-F and Bd3318-pBAD-R, using pBAD/gIIIA backbone	This study
pBAD-a11q_612	Expression of *B. exovorus* FliA, a11q_617, under arabinose inducible promoter in pBAD/gIIIA, constructed using primers A11q_617-pBAD-F and A11q_617-pBAD-R, using pBAD/gIIIA backbone	This study
pBBR1MSC2 pbd0119:lacZ	lacZ under *B. bacteriovorus flp*1 ,bd0119,promoter regulation, constructed using pBd0119-lacZ-F and pBd0119-lacZ-R, using pBBR1MCS2-LacZ backbone	This study
pBBR1MSC2 pbd0606::lacZ	lacZ under *B. bacteriovorus* flagellin, bd0606, promoter regulation, constructed using pBd0606-lacZ-F and pBd0606-lacZ-R, using pBBR1MCS2-LacZ backbone	This study
pBBR1MSC2 pbd3297::lacZ	lacZ under *B. bacteriovorus flp*3, bd3297, promoter regulation, constructed using pBd3297-lacZ-F and pBd3297-lacZ-R, using pBBR1MCS2-LacZ backbone	This study
pBBR1MSC2 pbd*4000*::lacZ	lacZ under *B. bacteriovorus flp*4, bd4000,promoter regulation, constructed using primers p*bd40004*-lacZ-F and p*Bd4000*-lacZ-R, using pBBR1MCS2-LacZ backbone	This study
pBBR1MSC2 pa11q_2366::lacZ	lacZ under *B. exovorus flp*1, A11q_2366, promoter regulation, constructed using pA11q_2366-F and p A11q_2366-lacZ-R, using pBBR1MCS2-LacZ backbone	This study
pBBR1MSC2 pa11q_1936::lacZ	lacZ under *B. exovorus* flagellin, A11q_1936, promoter regulation, constructed using pA11q_1936-F and p A11q_1936-lacZ-R, using pBBR1MCS2-LacZ backbone	This study
pBBR1MSC2 pa11q_630::lacZ	lacZ under *B. exovorus flp*3, A11q_630, promoter regulation, constructed using pA11q_630-F and p A11q_630-lacZ-R, using pBBR1MCS2-LacZ backbone	This study
pBBR1MSC2 pa11q_1811::lacZ	lacZ under *B. exovorus flp*4, A11q_1811, promoter regulation, constructed using pA11q_1811-F and p A11q_1811-lacZ-R, using pBBR1MCS2-LacZ backbone	This study
pPROBE-NT ::*flp1*-2	Complement bd0118-9, constructed using primers pPROBE-NT-bd0118-9-F and	This study

	pPROBE-NT-bd0118-9-R, using pPROBE-NT backbone	
pPROBE-NT ::*flp2*	Complement bd0118, constructed using primers pPROBE-NT-bd0118-F and pPROBE-NT-bd0118-9-R, using pPROBE-NT ::*flp1*-2backbone	This study
pPROBE-NT ::*flp1*	Complement bd0119, constructed using primers pPROBE-NT-bd0118-9-F and pPROBE-NT-bd0119-R, using pPROBE-NT backbone	This study
pPROBE-NT ::*flp4*	Complement *flp4*, constructed using primers pPROBE-NT-*flp4*-F and pPROBE-NT-*flp4*-R, using pPROBE-NT backbone	This study
pPROBE-NT::*flp1*-2,4	Complement *flp1*-2,*flp4*, constructed using primers pROBE-NT-*flp1*-2,4-F and pROBE-NT-*flp1*-2,4-R. This primmers amplified fp4 with its promoters, using pPROBE-NT *flp1*-2 as a backbone	This study
pPROBE-NT ::pbd0119-bd3297	Bd3297 induced by bd0119 promoter, constructed using primers Bd3297-pbd119-F and Bd3297-pbd119-F based on pPROBE-NT ::bd0119 plasmid.	This study
pSSK10 Δbd*flp1*-2	For knocking out the both *flp1* and *flp2*, constructed using two fragments, upstream and downstream to *flp*1-2, sawed together to pSSK10 plasmid. Fragments generated using primers pSSK-bd0118-9-F1, pSSK-bd0118-9-R1 (up fragment), pSSK-bd0118-9-F2 and pSSK-bd0118-9-R2 (down fragment).	This study
pSSK10 Δ*flp2*	For knocking out *flp2* constructed using two fragments, upstream and downstream to *flp*1-2, sawed together to pSSK10 plasmid Fragments generated using: pSSK10-bd0118-9-F1, pSSK10-bd0118-R1, pSSK10-bd0118-9-F2, pSSK-bd0118-9-R2	This study
pSSK10 Δ*flp1*	For knocking out *flp1*, constructed using fragment generated by using primers : pSSK10-bd0118-9-F1, pSSK10-bd0119-R1, pSSK10-bd0119-F2, pSSK-bd0118-9-R2	This study
pSSK10 Δ*flp3*	For knocking out *flp3*, constructed using fragment generated by using primers: Bd3297 knockout-F1, Bd3297 knockout-R1, Bd3297 knockout-F2, Bd3297 knockout-R2	This study
pSSK10 Δ*flp4*	For knocking out *flp4*, constructed using fragment generated by using primers: *flp4* knockout-F1, *flp4* knockout-R1, *flp4* knockout-F2, *flp4* knockout-R2	This study

Table S5. Primers used in this study

Primers	Sequence	Description
sqRT-bd0108-F	GCTTCTCCTTTGCGGGAACAG	Semi quantative PCR for bd0108
sqRT-bd0108-R	AGT CGC CCA AAG CCG ATG TC	Semi quantitative PCR for bd0108
sqRT-*flp*1-F	AGAATGAATCCGGCCAAGGTG	Semi quantitative PCR for bd0119
sqRT-*flp*1-R	GCCTGTGATATCACTTGCCAATG	Semi quantitative PCR for bd0119
sqRT-*flp*2-F	ATGACCGTAGAGTATGTGCTTT	Semi quantitative PCR for bd0118
sqRT- *flp*2-R	GTCCCAGCCAATGTGATTTCC	Semi quantitative PCR for bd0118
sqRT-*flp*3-F	GGACTTGTTGAGTACCTGATC	Semi quantitative PCR for bd3297
sqRT- *flp*3-R	GCTGAAGTCGCGCTTCTTG	Semi quantitative PCR for bd3297
sqRT- *flp4*-F	GAATAAACGAGGTCAAATCG	Semi quantitative PCR for bd4000
sqRT- *flp4*-R	AGATATTCTGCCATTTCGC	Semi quantitative PCR for bd4000
sqRT- *bd0199-F*	GGAAGAACTGACCCATCTGC	Semi quantitative PCR for bd0199
sqRT- *bd0199-R*	GAAGCTGCGAAGATCCTGAC	Semi quantitative PCR for bd0199
8f	AGAGTTTGATCCTGGCTCAG	Amplification of 16sRDNA, for identification of risidual DNA in RNA extraction
1429R	CGGTTACCTTGTTACGACTT	Amplification of 16sRDNA, for identification of risidual DNA in RNA extraction
pSSK10-*flp*1-2-F1	CCTTTTTAACCCATCACATATACCTGCCGT TGCCAGAATTCCCTGAACTCC	Forward primer for the amplification Bd0118-9 upstream region.
pSSK10-*flp*1-2-R1	GTTTCTCCGTCCCAGCCAATGTGATTTCCA TCAACAATAGCTTCTTGGAAAAGTTC	Reverse primer for the amplification Bd0118-9 upstream region..
pSSK10-*flp*1-2F2	TTGATGGAAATCACATTGGCTGG	Forward primer for the amplification Bd0118-9 downstream.
pSSK10-*flp*1-2-R2	CGTTTGGATTGCAACTGGTCTATTTTCCTC TTCCCACTTCTCTGTTAACTGC	Reverse primer for the amplification Bd0118-9 downstream.
pSSK10-*flp*2-R	CTCCGTCCCAGCCAATGTGATTTCCATCAA CGGTCATTGGGTCCCCCTTTTTATTC	Reverse primer for the amplification of the downstream region of the bd0118.
pSSK10-*flp*1-R1	GAAGCCTGTGATATCACTTGCCAATGAAC CCACCATGGATGTTCCTCCTTAGAGAC	Reverse primer for the amplification of the upstream, region of the bd0119.
pSSK10-*flp*1-F2	GTGGGTTCATTGGCAAGTGA	Forward primer for the amplification of the downstream region of the bd0119 operon.
pSSK10-*flp*3 -F1	CCTTTTTAACCCATCACATATACCTGCCGT T CCGCGACGACAACCAAATCC	Forward primer for the amplification of the upstream region of the bd3297
pSSK10-*flp*3 –R1	GCTGTCTGATTTCGAGTTGACGGAGCCTT CCATGATTTCTCCTGTGGCAGG	Reverse primer for the amplification of the upstream region of the bd3297
pSSK10-*flp*3 –F2	GAAGGCTCCGTCAACTCG	Forward primer for the amplification of the downstream region of the bd3297
pSSK10-*flp*3 –R2	CGTTTGGATTGCAACTGGTCTATTTTCCTC TTTCAATGAGTATCTGCCCTTTG	Reverse primer for the amplification of the downstream region of the bd3297
pSSK10-*flp*4 –F1	CCTTTTTAACCCATCACATATACCTGCCGT GAATCTGGTGGGCTTGCTTG	forward primer for the amplification of the upstream region of *bd4000*

pSSK10-*flp*4 –R1	CTCCACCACGATTTGACCTCG	Reverse primer for the amplification of the upstream region of *flp4*
pSSK10-*flp*4 –F2	CATGAATAAACGAGGTCAAATCGTGGTG GAGAAGAAGTAAACCCGAAAATTTTCAAA TC	forward primer for the amplification of the upstream region of *flp4*
pSSK10-*flp*4 –R2	CGTTTGGATTGCAACTGGTCTATTTTCCTC TCATGGACCTTTGCGTGGAATAC	Reverse primer for the amplification of the upstream region of *flp4*
pPROBE-NT-bd0118-9-F	GGAATTGGGGATCGGAAGCTTGCATGCC TG GACTGCACAATCACCATCAG	Forward primer for amplification of bd0119 promoter region
pPROBE-NT-bd0118-9-R	CAACTCCAGTGAAAAGTTCTTCTCCTTTAC TCATATTACTTGTTCTCGCGCAGCTG	Reverse primer for amplification of bd0118
pPROBE-NT-bd0118-F	TCCGATATTGTCTCTAAGGAGGAACATCC ATGACCGTAGAGTATGTGCTTTTAC	forward primer for amplification of bd0118
pPROBE-NT-bd0119-R	CAACTCCAGTGAAAAGTTCTTCTCCTTTAC TCATATCCAGCTAGACAGCGGAAATCG	Reverse primer for amplification of bd0119
Bd3297-pbd119-F	TATCCGATATTGTCTCTAAGGAGGAACAT CC ATGAAAGCTCAAACCATCATCAA	Forward primer for amplification of Bd3297
Bd3297-bpdb119-R	CAACTCCAGTGAAAAGTTCTTCTCCTTTAC TCATCTATTTGCTGTCTGATTTCGAGTTG	Reverse primer for amplification of Bd3297
pPROBE-NT-*flp4*-F	GGAATTGGGGATCGGAAGCTTGCATGCC TG AAACATCCTAGTGATGGTTTACC	Reverse primer for amplification of *flp4*
pPROBE-NT-*flp4*-R	CAACTCCAGTGAAAAGTTCTTCTCCTTTAC TCAGCAACGCTTTCCTTTGATTTG	Reverse primer for amplification of *flp4*
pROBE-NT-*flp1*-2,4-F	CCGTTGATGGAAATCACATTGGCTGGGAC GGAGAAACATCCTAGTGATGGTTTACC	Reverse primer for amplification of *flp4*
pROBE-NT-*flp1*-2,4-R	CCAATTGGAGTATTTTGTTGATAATGGTCT GCGCAACGCTTTCCTTTGATTTG	Reverse primer for amplification of *flp4*
Bd0119-8KO-check-F	TTTACGCCAGGGAGGTCCAGG	For validation *flp1*, *flp2* and *flp1*-2 knockout
Bd0119-8KO-check-R	TTGCCCAGATCAGAGCTCCCACC	For validation *flp1*, *flp2* and *flp1*-2 knockout
Bd3297-KO-check-F	GCTGCCAAGAAAGCTGAAGC	For validation *flp3* knockout
Bd3297-KO-check-R	CACGGATTCAGCACAGATCAGG	For validation *flp3* knockout
flp4-KO-check-F	CACATCAGCCCTCTGGAAGTAG	For validation *flp4* knockout
flp4-KO-check-R	ATTCGCTTCCTCGAACCAATCAC	For validation *flp4* knockout
Bd3318-pBAD-F	CCGTTTTTTGGGCTAACAGGAGGAATTAA CCATGGGGAAAAACGCGGCATTG	Forward primer for the amplification of the *B. bacteriovorus FliA (bd3318*)
Bd3318-pBAD-R	GGTCGACGGCGCTATTCAGATCCTCTTCT GTCATCAGGCGATTTCAAGCTCG	Reverse primer for the amplification of the *B. bacteriovorus FliA (bd3318*)
A11q_617-pBAD-F	CCGTTTTTTGGGCTAACAGGAGGAATTAA CCATGTCAAAAGCGAATCTTCTGAAGA	Forward primer for the amplification of the *B. exovorus FliA (a11q_617)*
A11q_617-pBAD-F	GGTCGACGGCGCTATTCAGATCCTCTTCT GTCATTAGATAGCTTCTAATTCGCCAGC	Reverse primer for the amplification of the *B. exovorus FliA (a11q_617)*
pBd0119-lacZ-F	GCGCGCGTAATACGACTCACTATAGGGC GAGACTGCACAATCACCATCAG	Forward primer for the amplification of *B. bacteriovorus flp*1, *bd0119*, promoter
pBd0119-lacZ-R	CGACGGCCAGTGAATCCGTAATCATGGTA GCCATGGATGTTCCTCCTTAGAG	Reverse primer for the amplification *of B. bacteriovorus flp*1, *bd0119*, promoter

pBd0606-lacZ-F	GCGCGCGTAATACGACTCACTATAGGGC GAGATGCGTGGTCATCACTCTCC	Forward primer for the amplification *of B. bacteriovorus* flagellin, *bd0606*, promoter
pBd0606-lacZ-R	CGACGGCCAGTGAATCCGTAATCATGGTA GCCATTTTGGGACCTCCATTAAATCG	Reverse primer for the amplification *B. bacteriovorus* flagellin, *bd0606*, promoter
pBd3297-lacZ-F	GCGCGCGTAATACGACTCACTATAGGGC GACTGAAGAAGTTCCGTCAAAGCC	Forward primer for the amplification *of B. bacteriovorus flp*3, *bd3297,* promoter
pBd3297-lacZ-R	CGACGGCCAGTGAATCCGTAATCATGGTA GCCATGATTTCTCCTGTGGCAGGAG	Reverse primer for the amplification *of B. bacteriovorus flp*3 ,*bd3297,* promoter
P*bd4000*-lacZ-F	GCGCGCGTAATACGACTCACTATAGGGC GA CCCTCTGGAAGTAGAGAAGTG	Forward primer for the amplification *of B. bacteriovorus flp*4 ,*bd4000,* promoter
P*bd4000*-lacZ-R	CGACGGCCAGTGAATCCGTAATCATGGTA GCCATGTTTAAGATTGTATCACACTTCC	Reverse primer for the amplification *of B. bacteriovorus flp*4 ,*bd4000,* promoter
pA11q_2366-lacz-F	GCGCGCGTAATACGACTCACTATAGGGC GAGCTAGGCCGTAACTACTCTGAT	Forward primer for the amplification *of B. exovorus flp*1 *,* a11q_2366*,* promoter
pA11q_2366-lacz-F	CGACGGCCAGTGAATCCGTAATCATGGTA GCCATATTCCACCCCTTTTACTGTG	Reverse primer for the amplification *of B. exovorus flp*1 *,* a11q_2366*,* promoter
pA11q_1936-lacz-F	GCGCGCGTAATACGACTCACTATAGGGC GAGAAAATGAAAGTTGCACTCTGGG	Forward primer for the amplification *of B. exovorus* flagellin *,* a11q_1936*,* promoter
pA11q_1936-lacz-F	CGACGGCCAGTGAATCCGTAATCATGGTA GCCATTGTAAAACTCCATTTTATTAAATTA TG	Reverse primer for the amplification *of B. exovorus* flagellin *,* a11q_1936*,* promoter
pA11q_630-F-lacZ-F	GCGCGCGTAATACGACTCACTATAGG GCGAGAAGCTTACGAAATTCTTGAGC	Forward primer for the amplification *of B. exovorus flp*3 *,* a11q_630*,* promoter
pA11q_630-F-lacZ-R	CGACGGCCAGTGAATCCGTAATCATG GTAGCCATAGACGACCTCACTCCG	Reverse primer for the amplification *of B. exovorus flp*3 *,* a11q_630*,* promoter
pA11q_1811-F-lacZ-F	GCGCGCGTAATACGACTCACTATAGGGC GAAACATGGCAGCAACGTATTCC	Forward primer for the amplification *of B. exovorus flp*4 *,* a11q_1811*,* promoter
pA11q_1811-F-lacZ-R	CGACGGCCAGTGAATCCGTAATCATGGTA GCCATGTGTTTTAGCTTTTCCAACC	Reverse primer for the amplification *of B. exovorus flp*4 *,* a11q_1811*,* promoter
fliAlambdared-F	GATAACTCATATAACGCAGGGCTGTTTAT CGTGAATTCACTCTATACCGCTGGTGTAG GCTGGAGCTGCTTC	Forward primer for constriction liner fragment for *E. coli* FliA inactivation
fliAlambdared-F	GTCAAAGTTAAAGTGCGGCATTTACTGAC GTTATAACTTACCCAGTTTAGTGATGGGA ATTAGCCATGGTCC	Reverse primer for constriction liner fragment for *E. coli* FliA inactivation
Colifliadel-F	GCAGAAACGGATAATCATGCC	Forward primer for validation of *E. coli* inactivation
Colifliadel-R	CACCATCATTAAGAACTCCTGG	Reverse primer for validation of *E. coli* inactivation

References

1. Karunker, I., et al., *A global transcriptional switch between the attack and growth forms of Bdellovibrio bacteriovorus.* PLoS One, 2013. **8**(4): p. e61850.
2. Fitzgerald, D.M., R.P. Bonocora, and J.T. Wade, *Comprehensive mapping of the Escherichia coli flagellar regulatory network.* PLoS Genet, 2014. **10**(10): p. e1004649.
3. Omotajo, D., et al., *Distribution and diversity of ribosome binding sites in prokaryotic genomes.* BMC Genomics, 2015. **16**: p. 604.
4. Li, W., et al., *The EMBL-EBI bioinformatics web and programmatic tools framework.* Nucleic Acids Res, 2015. **43**(W1): p. W580-4.
5. Crooks, G.E., et al., *WebLogo: a sequence logo generator.* Genome Res, 2004. **14**(6): p. 1188-90.
6. Roschanski, N., et al., *Identification of genes essential for prey-independent growth of Bdellovibrio bacteriovorus HD100.* J Bacteriol, 2011. **193**(7): p. 1745-56.
7. Simon, R., U. Priefer, and A. Puhler, *A Broad Host Range Mobilization System for In Vivo Genetic Engineering: Transposon Mutagenesis in Gram Negative Bacteria.* Nat Biotech, 1983. **1**(9): p. 784-791.
8. Ferrieres, L., et al., *Silent mischief: bacteriophage Mu insertions contaminate products of Escherichia coli random mutagenesis performed using suicidal transposon delivery plasmids mobilized by broad-host-range RP4 conjugative machinery.* J Bacteriol, 2010. **192**(24): p. 6418-27.
9. Fried, L., J. Lassak, and K. Jung, *A comprehensive toolbox for the rapid construction of lacZ fusion reporters.* J Microbiol Methods, 2012. **91**(3): p. 537-43.
10. Miller, W.G., J.H. Leveau, and S.E. Lindow, *Improved gfp and inaZ broad-host-range promoter-probe vectors.* Mol Plant Microbe Interact, 2000. **13**(11): p. 1243-50.
11. Steyert, S.R. and S.A. Pineiro, *Development of a novel genetic system to create markerless deletion mutants of Bdellovibrio bacteriovorus.* Appl Environ Microbiol, 2007. **73**(15): p. 4717-24.
12. Datsenko, K.A. and B.L. Wanner, *One-step inactivation of chromosomal genes in Escherichia coli K-12 using PCR products.* Proc Natl Acad Sci U S A, 2000. **97**(12): p. 6640-5.
